# Maternal undernutrition reprograms reproductive and metabolic phenotypes in female offspring of the rabbit model

**DOI:** 10.1371/journal.pone.0345066

**Published:** 2026-03-26

**Authors:** María Arias-Alvarez, Alfonso Gutiérrez-Adan, Eva Pericuesta, Pablo Bermejo-Alvarez, Pilar Millán, María Rodríguez, Pedro Luis Lorenzo, Pilar García Rebollar, Rosa María García-García

**Affiliations:** 1 Department of Animal Production, Veterinary Faculty, Complutense University of Madrid, Madrid, Spain; 2 Department of Animal Reproduction, National Institute of Agricultural and Food Research and Technology, Higher Council for Scientific Research, Madrid, Spain; 3 Department of Physiology, Veterinary Faculty, Complutense University of Madrid, Madrid, Spain; 4 Department of Agrarian Production, Higher Technical School of Agricultural, Food and Biosystems Engineering, Technical University of Madrid, Madrid, Spain; Justus Liebig Universitat Giessen, GERMANY

## Abstract

Maternal undernutrition during pregnancy in mammals can induce long-term effects in offspring health through molecular programming of the gametes. Using the rabbit model, this study investigates whether maternal food restriction (FR) during the first two-thirds of gestation induces effects on ovarian follicular, oocyte, and early embryo developmental markers in F1 female offspring at the onset of reproductive life. Additionally, body composition, metabolic profile, and growth trajectory from birth to sexual maturity (16 weeks) were assessed to evaluate potential impacts on overall health. Pregnant females (F0) were fed either *ad libitum* (Control group) or a restricted diet covering 60% of nutritional requirements (FR group). Offspring from both groups were fed *ad libitum*. Maternal FR had no significant effects on birth weight and survival of progeny, growth trajectory, feed intake or glycemic profile during the juvenile phase. Body weight, body composition, lipid, and glycemic profiles in F1 sexually mature females were similar. However, serum aminotransferase levels were significantly elevated in the F1 females from FR group (P < 0.05), indicating potential hepatic stress. In FR group, F2 oocytes showed a significant upregulation of *SOD2, G6PD*, and *FABP4* mRNA expression levels (P < 0.05), while cumulus cells (CCs) exhibited increased *TP53* and decreased *CASP3* transcripts levels (P < 0.05). At ovulation time, the progesterone/estradiol ratio was significantly higher (P < 0.001), coinciding with an increased proportion of F2 expanded blastocysts (P < 0.005) and total embryo cell counts (P < 0.05). Serum anti-Müllerian hormone levels, ovulation rate, apoptosis rate, and *in vitro* embryo development did not differ between groups. These findings suggest that re-feeding after maternal food restriction can attenuate adverse long-term effects on offspring growth and metabolism, while modulating the expression of genes related to oxidative stress, apoptosis and fatty acid uptake in the oocytes of F1 females. This modulation may reflect the activation of compensatory intracellular mechanisms that support early embryonic development in juvenile females at the onset of their reproductive life.

## Introduction

According to the concept of Developmental Origins of Health and Disease (DOHaD), early life events can induce permanent structural and functional modifications in foetal organs preparing the organism for predicted postnatal conditions [1]. This framework is supported by extensive evidence showing that maternal nutrition during pregnancy plays critical role in organogenesis, and metabolic programming, thereby influencing the offspring’s long-term health and predisposing to chronic diseases such as obesity, and type 2 diabetes through epigenetic mechanisms [2–7]. The thrifty gene hypothesis proposed by James Neel [8] posits that in utero undernutrition activates adaptive genetic responses —such as genes involved in fat storage capacity, glycolysis or energy metabolism—beneficial to ensure fetal survival, but detrimental when nutrition is abundant in postnatal life [9].

The influence of the nutrition of women and adolescent girls of reproductive age on the health and reproductive potential of their progeny represents a critical public health concern [10]. Epidemiological studies of the Dutch winter famine, during which caloric intake was reduced by 60–70%, demonstrated that maternal malnutrition during pregnancy can induce long-term health consequences in the offspring, even in the absence of marked effects on birth weight [11]. Currently, an estimated 29.3% of the global population (approximately 2.3 billion people) experiences moderate to severe food insecurity [12], with an increase of 150 million people affected by hunger since the onset of the COVID-19 pandemic [13]. Calorie restriction diets -typically involving a reduction of 30–50% of daily maintenance requirements- are common among women of reproductive age in both high- and low-income countries, albeit driven by different socioeconomic and cultural factors.

Nutritional requirements vary throughout pregnancy, particularly during critical developmental periods [14], such as the formation and proliferation of fetal germ cells, which are particularly vulnerable to nutritional disturbances [5,15]. Epigenetic modifications to the genome that arise during fetal development in the germ line can persist in future generations by altering the gene expression in oocytes and therefore, affect fertility across F1 and F2 generations through their effects on oocyte competence and early embryo development [10,16]. Altered DNA methylation in F1 oocytes may transmit these metabolic disturbances to the next generation, as such epigenetic changes persist in adult F2 tissues [17]. Many studies in animal models, such as rats, mice, and humans, have suggested various epigenetic factors (e.g., DNA methylation, histone modification, and small RNAs) in germ cells may carry these memories [18,19]. The female offspring are more affected by in utero malnutrition than males [20]. As a consequence, some studies have demonstrated that acute fasting before ovulation compromises oocyte developmental potential in rabbits [21], while prolonged caloric restriction alters the transcriptomic profile in mice [22]. However, the effects of maternal food restriction during gestation on the transcriptional profile of oocytes from F1 females remain largely unexplored [23].

Prenatal maternal dietary restriction to 50% of nutrient requirements has been associated with impaired ovarian offspring development [2,24], reduced follicular reserve [25–28], altered follicular steroidogenic activity [29], disrupted ovulation, and increased follicular atresia in sheep, cow and rodents models [30]. Nevertheless, early-life adaptations leading to long-term reproductive dysfunctions may depend on several factors, including the timing and severity of the nutritional insult, as well as species-specific physiological responses [24,25,31–33]. Both the amount of food consumed, and the composition of the diet are important factors [34]. Interestingly, recent studies have challenged the prevailing notion of detrimental maternal food restriction effects, reporting no adverse consequences on ovarian and uterine function or fertility in heifers exposed to prenatal or postnatal nutritional interventions [35,36]. These findings are particularly relevant for elucidating the role of nutrition in reproductive health and may offer valuable translational models for both human and animal reproductive biology.

Ovarian oxidative stress is suggested as a possible mechanism to explain the changes in offspring reproduction in response to maternal undernutrition [25,29,37]. Oxidative stress induces a cascade of chain reactions that can ultimately lead to lipid peroxidation in membrane phospholipids, affecting the function and permeability of cell membranes, and generating DNA damage, meiotic arrest and mitochondrial dysfunction, which, in turn, activate caspase cascades and culminate in irreversible cell death [38]. Given that mitochondria are inherited exclusively through the maternal line, such alterations may compromise oocyte competence and early embryonic development [39] and negatively impact the fertility potential of female progeny [40]. Rabbits are a valuable translational model for human health research, particularly for investigating the mechanisms regulating fetal growth, developmental reprogramming, and their long-term physiological consequences [23,41–44]. Compared to rodents, rabbits exhibit higher biochemical and physiological similarity to humans [45,46], and are considered a more appropriate model for studying human lipid metabolism [47]. Furthermore, their genome is more closely related to that of humans [48], and their extensive historical use in toxicology has led to a well-characterized physiology [10,49,50]. As induced ovulators, rabbits also offer the advantage of precise control over the timing of ovulation, embryo development, and gestation. The use of artificial insemination (AI) further reduces variability by minimizing confounding paternal effects.

**Fig 1:**
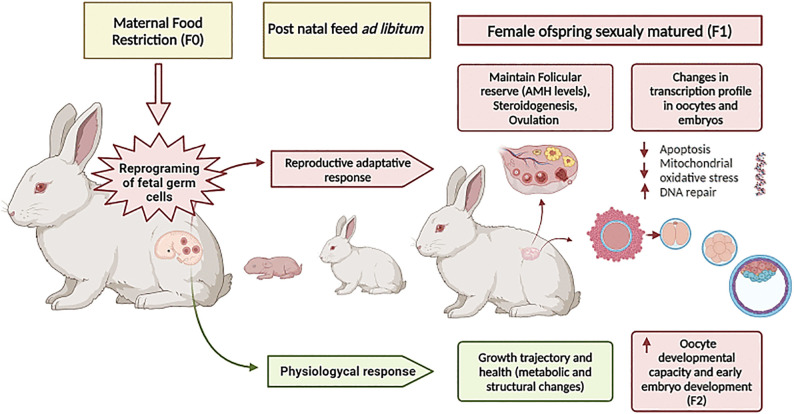
Graphical abstract. Summary of the experimental design and key findings of this study.

The main aim of this study was to test the hypothesis that 40% of food restriction (FR) during early and mid-pregnancy (covering 60% of nutritional requirements) exerts long-term effects on F1 female offspring, particularly on ovarian function (ovulation rate, follicular reserve, and steroidogenic activity), oocyte competence, and embryo development. We used a well-established rabbit model of maternal FR during the first two thirds of gestation [41–44,50], consistent with the definition of FR by the World Health Organization [13]. This period encompasses key developmental stages, including preimplantation (days 0–7), organogenesis and gonadal differentiation (days 14–16), and placental formation (days 10–17) [50]. Previous studies demonstrated that this model alters lipid metabolism during early to mid-gestation [41], while maintaining maternal energy homeostasis (glucose and insulin levels) and lipid metabolism (NEFA) at term. Compensatory feeding in late gestation mitigates maternal body weight and body reserve losses in this rabbit model [42]. To estimate ovarian reserve, serum anti-Müllerian hormone (AMH) concentrations were measured as a reliable biomarker. Specific target genes involved in metabolic processes (fatty acid uptake, transport and energy metabolism); redox balance and mitochondrial function, cell cycle regulation and apoptosis, meiotic progression, and Cumulus Cells (CCs) steroidogenesis and expansion were evaluated as potential markers of oocyte and embryo quality in F1 offspring at the onset of reproductive life (Fig 1).In addition, given the known long-term impact of maternal FR on offspring health and metabolism, we assessed postnatal and juvenile growth, feed intake, and metabolic parameters (glycemic, lipid, and hepatic profiles) and body composition in sexually mature F1 females to provide integrative insights into their reproductive performance.

## Materials and methods

Unless otherwise stated, all chemicals were purchased from Merk (Spain).

### Animals and experimental design

New Zealand White x California rabbit does (*Oryctolagus cuniculus*) were housed individually in flat-deck cages under conditions of constant light/darkness (16:8 h), a temperature of 20–25ºC and relative humidity of 60–75% using a forced ventilation system. Animals had free access to fresh water. This study was performed according to Spanish Policy for Animal Protection RD53/2013. All experimental procedures with animals were approved by the Animal Ethics Committee of the Madrid Polytechnic University (UPM, Spain) and approved by the competent authority in animal welfare (code of project: PROEX 302/15) and complied with Spanish guidelines for the care and use of animals in research, according to European Union Regulation 2010/63/UE.

### Maternal food restriction model (F0 generation)

Multiparous females of 6 months of age (F0) were fed *ad libitum* a commercial diet that meet the nutritional requirements of rabbits, especially regarding calories and protein content (2400 kcal/kg of digestible energy, 16% crude protein, 37% crude fibre, and 3.7% fat, NANTA, Madrid, Spain). Diet was formulated following the nutritional recommendations for breeding does by de Blas and Mateos [51]. There was only one diet, provided in different quantities for the experimental groups. Feed intake was recorded daily and established in a mean of 175 g per animal and day. The animals did not have access to bedding as an additional nutrient source during the period of FR.

**Fig 2:**
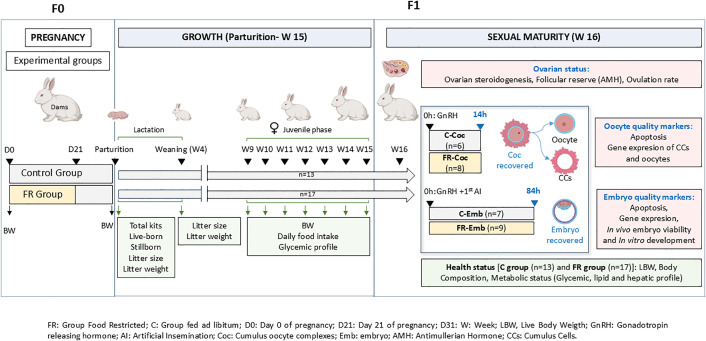
Experimental design. Pregnant females (F0) were fed *ad libitum* all through the gestation period (group C) or were feed restricted (105 g/day) from Day 0 (artificial insemination, AI) to Day 21 (group FR) of gestation (gestation length is 31 days). In female offspring (F1), sampling was specially focused when does initiate their reproductive life (16 weeks). The factors examined were body weight (BW), body composition and metabolic status. After induced ovulation with GnRH analogue treatment, ovarian status and, oocyte and embryo quality markers were examined 14 h later (Coc groups) or 84 h after ovulation induction and AI (Emb groups). Also, offspring performance was assessed at parturition, weaning (week 4) and from week 9 to 15 (juvenile phase). Female offspring were fed *ad libitum* all their postnatal life. FR: feed restriction group; C: control ad libitum feeding group; D0: Day 0 of pregnancy; D21: Day 21 of pregnancy; D31: Day 31 of pregnancy (delivery); LBW, live body weight; GnRH: gonadotropin-releasing hormone; AI: artificial insemination; Coc: cumulus oocyte complex; Emb: embryo; AMH: antimullerian hormone; CCs: cumulus cells.

All F0 females were artificially inseminated (AI) using a pool of fresh diluted semen with more than 20 million spermatozoa per dose in 0.5 mL of a commercial diluent (commercial extender MA-24 Ovejero, León, Spain). Ovulation was induced by intramuscular injection of 20 μg gonadotropin-releasing hormone (Inducel-GnRH, Ovejero, León, Spain). At this stage, females were randomly assigned to two experimental groups: the control group (C, n = 72) which received the standard diet *ad libitum* throughout pregnancy, and the food restriction group (FR, n = 70), which received a quantitatively restricted diet (Fig 2). The FR females were fed 60% of their previous daily food intake (105 g/day) until day 21 of pregnancy, followed by *ad libitum* feeding for one week more until the end of pregnancy (gestation length is usually 31 days) according to our previous experimental design of moderate maternal FR [42–44]. A pregnancy diagnosis was made by abdominal palpation. Maternal body weight (BW) from 11 C and 15 FR pregnant does randomly chosen was recorded on the day of the AI and at the end of pregnancy.

### Female offspring (F1 generation)

At birth, the total number of kits, number of live-born, and stillborn were recorded from 41 C and 49 FR females. The number of kits per litter was adjusted from 7 to 12at the beginning of lactation. Litter size and total litter weight were recorded both at parturition and weaning (4 weeks of age). Offspring were continuously fed *ad libitum* with the same control diet as their F0 dams until reaching sexual maturity at 16 weeks of age. From 9 weeks to 15 weeks of age (juvenile stage), a total of 13 C and 17 FR daughters (F1 generation) were randomly chosen from F0 dams belonging to control and FR group. These F1 females were housed in individual cages, and their BW was monitored weekly, while feed intake and glycemic profile were assessed every two weeks. When F1 females reached sexual maturity (at 16 weeks of age), BW, body composition (BC), blood metabolic profile (glycemic, lipid and hepatic markers), ovarian steroidogenic response (estradiol and progesterone) and estimated ovarian follicular reserve by measuring serum levels of Anti-Müllerian hormone (AMH) were assessed. Blood samples were obtained from the ear vein in tubes with anticoagulant, centrifuged for 15 min at 700 g at 4 ºC, and plasma was stored at −80 ºC until analysis. All samples were collected at 09:00 h to avoid the effects of circadian variations.

F1 females of each experimental group (C and FR) were randomly divided into two subgroups: 1) Group Coc, for the study of cumulus-oocyte complex (COC) quality markers (subgroups C-Coc, n = 6, and FR-Coc, n = 8) and, 2) Group Emb, to assess embryo quality markers and *in vivo* and *in vitro* embryo developmental capacity (subgroups C-Emb, n = 7 and FR-Emb, n = 9). In Group Coc, ovulation was induced by the intramuscular injection of GnRH (20 μg; Inducel-GnRH; Ovejero) (as described before for F0 females). In the Emb groups, ovulation was also induced, and AI was performed simultaneously, as previously described for the F0 females. Reproductive tracts were obtained by mid-ventral laparotomy after sedation with 35 mg/kg of ketamine (Imalgene; Merial), and euthanasia using an intravenous bolus of barbiturate (30 mg/kg; Dolethal; Vetoquinol). Ovulated COCs were recovered by flushing the reproductive tract 14h after in both experimental groups (C-Coc and FR-Coc) [52]. Embryos were recovered 84h after GnRH administration + AI in both experimental groups (C-Emb and FR-Emb) [53]. Oviducts were flushed with phosphate-buffered saline (PBS) supplemented with 0.1% BSA (w/v). The rates recorded were ovulation rate (number of corpora lutea (CL)/ doe), COC recovery rate (COCs/ number of recorded CL x 100), and embryo recovery rate (embryos/ CL counts x 100). Embryo morphology was assessed immediately after their recovery. Expanded blastocysts were fixed in 4% paraformaldehyde for the apoptosis study, and the embryo cell counting. Early blastocysts were immediately snap-frozen in liquid nitrogen and storage at −80 °C for transcript abundance analysis by quantitative real-time polymerase chain reaction (qRT-PCR). The remaining morphologically viable embryos were *in vitro* cultured (IVC) to establish their developmental potential [54]. Embryos were pooled per individual F1 female and the animal was considered the experimental unit for gene expression analyses.

### Estimation of body composition

The BC was determined at week 16 in F1 females by bioelectrical impedance analysis (BIA). A four-terminal body composition analyser (Model Quantum II, RJL Systems, Detroit, MI, USA) was used to determine bioelectrical resistance and reactance (both in ohms) according to [55]. Impedance, which represents the opposition encountered by the electrical current as it passes through the animal’s body, was then calculated as follows:

Impedance = (resistance² + reactance²)^½^. The regression equations described by [56] were then used to estimate water, protein, ash, fat, and energy contents in relation to BW, parity number, and physiological status of the rabbit.

### Evaluation of metabolic and hormonal status

#### Glycemic, lipid and hepatic profiling.

We assessed serum parameters related to the offspring’s long-term health that can affect reproduction, including glycemic metabolism (glucose, insulin, and glucose/insulin ratio), lipid metabolism (total cholesterol, triglycerides (TG) and TG/cholesterol ratio), and hepatic profile (aminotransferase (AST), serum alanine transaminase (ALT) and AST/ALT ratio). All ratios between two metabolites were calculated by dividing the serum concentrations of one metabolite by that of the other for each animal, and then the mean value was calculated for each experimental group.

Carbohydrate/lipid metabolism and hepatic variables were measured using commercial kits (Biolabo, Maizy, France) in a clinical biochemical analyser (Tecom Science Co., Jiangxi, China) according to the procedures described by [42].

Plasma insulin concentrations were determined with a rabbit insulin ELISA kit (Mercodia AB, Uppsala, Sweden). The assay sensitivity of this method was 0.15 mUI/L, the intra-assay variation coefficient was 3.9% and inter-assay variance was 5.30%. Insulin resistance was identified through homeostasis model assessment (HOMA-IR) according to [33] and calculated using the equation [insulin (mU/L) × (glucose (mg/dL)/18)]/ 22.5.

#### Anti-müllerian hormone levels.

AMH concentrations were determined in duplicate samples using a specific rabbit AMH competitive ELISA kit (MyBioSource, San Diego, CA, USA). Absorbances were measured using an automatic plate reader (Atat Fax 3200, Awareness Technology INC) at 450 nm. Intra-assay and inter-assay coefficients of variation were below 10%. The detection limit after adjusting the standard curve to rabbit values was 0.1 ng/mL.

#### Ovarian steroidogenic response.

Serum estradiol (E2) and progesterone (P4) concentrations were analysed in duplicate samples using a competitive ELISA kit (DE4299 and DE1561, Demeditec Diagnostics GmbH, Kiel, Germany), as previously reported [54]. Absorbances were measured using an automatic plate reader (Atat Fax 3200, Awareness Technology INC) at 450 nm. Intra-assay and inter-assay coefficients of variation were 6.3% and 7.6% for E2, and 6.4% and 6.6% for P4, respectively. Detection limits were 1.4 pg/mL (E2) and 0.045 ng/mL (P4).

### Quantitative gene expression study in cumulus oocyte complexes and blastocysts

#### RNA extraction and reverse transcription.

For transcript abundance analysis, we used 41 and 56 COCs (C-Coc and FR-Coc, respectively), and 32 and 40 early blastocysts (C-Emb and FR-Emb, respectively). CCs were mechanically removed by gentle repeated pipetting in phosphate buffered saline (PBS) + 1 mg/mL polyvinylpyrrolidone (PVP) medium. Groups of 10–12 blastocyst and 10–12 oocytes and their corresponding CCs were analysed separately. Pools of blastocysts, oocytes and CCs were placed in different 1.5 mL Eppendorf tubes, snap-frozen in liquid nitrogen, and stored at −80ºC until analysis. For RNA extraction and reverse transcription, poly (A) RNA was prepared as described previously [52,57,58] using the Dynabeads mRNA Direct Extraction KIT (Dynal Biotech, Thermo Fisher Scientific, Waltham, MA, USA) according to the manufacturer’s instructions with minor modifications. The reverse transcription (RT) reaction (Bioline, Thermo Fisher Scientific) was performed using poly (T) primer, random primers, and murine Moloney leukemia virus reverse transcriptase enzyme (M-MLV High-performance Reverse Transcriptase; Epicentre Biotechnologies, Thermo Fisher Scientific) in a total volume of 40 mL to generate cDNA. First, tubes were heated at 70ºC for 5 min to denature the secondary RNA structure, and then the RT was completed with the addition of 100 units of Superscript M-MLV RT enzyme. The samples were subsequently incubated at 42ºC for 60 min to allow for RT of RNA, followed by incubation at 70ºC for 10 min to denature the RT enzyme.

#### Quantitative real-time polymerase chain reaction.

Messenger RNA transcripts were quantified through qRT-PCR. Experiments were conducted to determine levels of each transcript relative to that of the housekeeping gene *H2AFZ* (histone family member z) previously validated for rabbit oocytes [52,59] and embryos [21,53,57,60]. All primers were designed using Primer–BLAST software (http://www.ncbi.nlm.nih.gov/tools/primersblast) to span exon-exon boundaries when possible. All qPCR reactions were performed in duplicate in a Rotorgene 6000 RealTime Cycler (Corbett Research Ltd, Cambridge, UK) after the addition of 2 µL samples to the PCR mix (GoTaq qPCR Master Mix; Promega Thermo Fisher Scientific) containing specific primers to amplify the housekeeping gene; genes related to important events in oocyte maturation and early embryo development in rabbits and markers of quality based on previous studies [21,52,53,57,59,60], namely *TFAM, PRDX3, SOD2* (mitochondrial function and redox balance), *G6PD* (defence against oxidizing agents and meiotic progression), *TP53* and *CASP3* (cell cycle regulation and apoptosis related pathways), *SLC274A, FABP4, CPT1* (fatty acid uptake and transport proteins), *GAPDH* (glycolysis) were used. In addition, *GJA1* and *CYP19A1* were quantified only in CCs because their expression is related to cell-to-cell communication for cumulus expansion in the COC, and steroidogenic secretion, respectively, and therefore they are indirect markers of oocyte competence. Primer sequences, approximate sizes of the amplified fragments, and melting temperatures for all transcripts are provided in Table 1. Cycling conditions were 94ºC for 3 min, followed by 35 cycles of 94ºC for 15 s, 56ºC for 30 s, 72ºC for 10 s, and 10 s at the corresponding melting temperature for each primer before fluorescence acquisition. Each pair of primers was previously validated [21,52,53,57,59,60] and optimized to achieve efficiencies close to 1. The comparative cycle threshold (CT) method was then used to quantify expression levels as described by [61]. To avoid primer-dimer artefacts, fluorescence was acquired in each cycle at a temperature higher than the melting temperature of primer dimers (specific for each product; 76–86ºC). Next, the threshold cycle or the cycle during the log-linear phase of the reaction at which fluorescence increased above the background was determined for each sample. Quantification was normalized against that of the endogenous control *H2AFZ*. According to the comparative CT method, the ΔCT value was determined by subtracting the *H2AFZ* CT value for each sample from each gene’s CT value. To calculate ΔΔCT, we used the highest sample ΔCT value as an arbitrary constant to subtract from all other ΔCT sample values. Fold changes in the relative gene expression of the target were determined using the formula 2^-ΔΔCT^.

**Table 1:** Primers used to amplify genes of interest in rabbit oocytes, cumulus cells, and blastocysts by quantitative reverse transcription–polymerase chain reaction.

Gene	Primer sequence (50–30)	Fragment size (bp)	Melting Temp. (ºC)	GenBank Accession no.
***H2AZ1***	F: AGGACGACCAGCCACGGACGTGTG R: CCACCACCAGCAATTGTAGCCTTG	208	84	XM_051820147
***TFAM***	F: TCCACCTTTGGCAGCTATCC R: CAGGAAGTTCCCTCCACAGC	148	80	XM_017348769.1
***PRDX3***	F: ATGTGAACTGTGAAGTTGTCGC R: TCTGCTTGGTTAAGTCCGACAG	132	85	XM_002718686.3
***SOD2***	F: ACTTGCAGATTGCTGCGTGT R: CAGGTAGTAAGCGTGTTCCC	100	80	XM_051854201
***TP53***	F: GTGCTGACCAGGGACACGGC R: CTGCACCAGGGCAGACCAGC	223	85	NM_001082404.1
***CASP3***	F: CATCGAGACAGACAGTGGG R: AGGGACTGGATGAACCAGGA	143	80	NM_001082117.1
***SLC27A4***	F: CCCTGGTGTACTATGGATTCCG R: GAACTTCTTCCGGATCACCACT	140	85	XM_002722970.3
***FABP4***	F: CGAGATTTCCTTCAAACTGGGC R: CATTCCACCACCAGTTTATCGC	171	86	XM_002710655.3
***CPT1A***	F: TGGACGTGGGAAGAACAAGCAG R: TGTCGAAACACCTGCCGTGCAG	155	83	NW_026256938.1
***G6PD***	F: CTGTCCAACCACATCTCCTCC R: TCCCGGATGATGCCAAATTCA	226	82	NM_001171382
***GAPDH***	F: CGCTGGGACGGGGTGCCCTTCATC R: CGCCAGGCCTCCCGCAGTTCATCA	347	80	XM_583628.4
***GJA1***	F: TGCCTTTCGTTGTAACACTCA R: AGAACACATGAGCCAAGTACA	142	80	NM_001198948.1
***CYP19A1***	F: GTGGATGTGCAGGGAAGTACA R: GTCACTGGTCTCATCTGGGTG	155	80	NM_001170921.2

*H2AFZ1* (histone family member z), *TFAM* (mitochondrial transcription factor A), *PRDX3* (peroxiredoxin 3), *SOD2* (superoxide dismutase 2, mitochondrial), *TP53* (tumour protein 53), *CASP3* (caspase 3, apoptosis-related cysteine protease), *SLC274A* (solute carrier family 27 member 4), *FABP4* (fatty acid binding protein 4), *CPT1A* (carnitine palmitoyltransferase 1A), *G6PD* (glucose-6-phosphate dehydrogenase), *GAPDH* (glyceraldehyde-3-phosphate dehydrogenase), *GJA1* (gap junction protein, alpha 1), *CYP19A1* (cytochrome P450 family 19 subfamily A member 1).

### Apoptosis assessment in cumulus oocyte complexes and blastocysts, and quantification of embryo cell number

The subgroups of COCs (C-Coc, n = 11; FR-Coc, n = 14) and blastocyst (C-Emb, n = 16; FR-Emb, n = 27) were pretreated with 20 mg/mL proteinase K working solution for 1 h in a humidified dark chamber at 37ºC. Strand breaks in DNA were detected by the terminal deoxyribonucleotidyl transferase-mediated dUTP–digoxigenin nick end-labelling method (TUNEL; In Situ Cell Death Detection Kit, POD; Roche Diagnostics) following a protocol adapted from [52,59]. Positive control sections were treated with DNAse I for 10 min at room temperature (RT) in a humidified chamber before incubation for the TUNEL reaction. As a negative control, samples were incubated with the label solution of the TUNEL reaction mixture without the enzyme solution. To avoid RNA interference, COCs and embryos were treated with RNAses before staining. Finally, they were counterstained with 0.25 mg/mL propidium iodide for 15 min and mounted between a coverslip and glass slide in mounting solution (ProLong Gold Antifade Reagent, Invitrogen, Waltham, MA, USA). Samples were observed under a laser scanning confocal microscope (Leica TCS SP2, Wetzlar, Germany) using a 488-nm excitation laser to visualize TUNEL-positive cells, and a 546-nm excitation laser to assess red fluorescence. Format, laser, gain, and offset were kept constants for all samples. Images were taken every 5 µm in z axis and then analysed using ImageJ software (http://rsbweb.nih.gov/ij/). The apoptosis index was calculated as the ratio of green area (TUNEL positive) divided by the red area (PI-positive) x100. Maximal projection was obtained with all sections taken in each COC to visualize the whole apoptotic area. In red nuclei (PI-positive) in embryos were quantified using the FindStackMaxima macro, which automates the counting process. Embryo quality was determined as the total number of cells per blastocysts within each experimental group.

### *In vivo* and *in vitro* embryo developmental competence

Embryo development was assessed by examining morphology immediately after recovery following the International Embryo Transfer Society (IETS) guidelines and according to their developmental stage (https://www.iets.org/) using a stereomicroscope (SMZ 800, Nikon, Japan). Early and expanded blastocyst were morphologically classified based on blastocoel expansion and following the guidelines described by Bo and Mapletof [62]. Rates of recovered blastocysts and degenerated or retarded embryos *in vivo* (embryos whose stage of development did not correspond to time after ovulation, i.e.,: zygotes, 2–16 cell embryos, and early morulae) were calculated relative to the total number of recovered embryos.

The ability of recovered embryos to develop *in vitro* was assessed by *in vitro* culture (IVC). Only morphologically good quality embryos recovered (n = 17, in C group and n = 22 in FR group) were cultured in groups (5–10 embryos/group) in TCM-199 supplemented with 10% foetal calf serum (FCS) at 38ºC under a 5% O_2_ and 5% CO_2_ atmosphere in air at maximum humidity according to a procedure described elsewhere [52,59]. Embryo developmental competence was determined by assessing their development to the expanded blastocyst stage in 48 h under a stereomicroscope (SMZ800; Nikon, Tokyo, Japan), as previously reported [54]. The embryo viability rate after IVC was obtained by dividing the number of blastocysts by the number of cultured embryos.

### Statistical analysis

Data were analysed using SAS software (9.4, Enterprise edition, SAS) and SPSS Version 19 (IBM SPSS Statistics). The Shapiro–Wilks test was used to assess normality and homogeneity of variance.

In dams (F0), evolution of body weight was assessed by repeated measures analysis (MIXED procedure). The model included the time (AI and birth) and their interaction as main effects. The dam was considered the experimental unit.

In litters (F1), total number of kits at parturition (live + stillborn), number of live-born kits, number of stillborn kits, litter weight at birth, as well as litter size and weight at weaning (number of kits), litter weight at weaning, were analysed by GLM procedure with FR as the main source of variation. The number of kits per litter, ranging from 7 to 12, was included as a covariate at the beginning of lactation. The litter was considered the experimental unit.

In the juvenile stage (F1), the evolution of BW in F1 offspring, feed intake and blood glucose concentrations were assessed by repeated measures analysis (MIXED procedure). The model included the FR, the age of the rabbits and their interaction as main effects. Each daughter (F1) was considered the experimental unit.

At 16 weeks old (F1), BW, estimated BC, serum metabolic and endocrine parameters, and number of CL (ovulation rate) were analysed as a completely randomized design, with FR as the main source of variation, using the GLM procedure. To compare serum steroidogenic hormones between experimental groups and at both time points (14 h and 84 h after ovulation), a Wilcoxon test for non-parametric samples was used. Each daughter (F1) was considered the experimental unit.

For apoptosis rate, embryo cell count, and mRNA transcript expression, an analysis of variance was performed considering only the FR as the main effect. Morphological categorization of recovered embryos between experimental groups and *in vitro* early embryo development was compared using Wilcoxon’s rank sums test. To assess *in vitro* embryo development over time, a Friedman test was performed.

Grubbs’ test was used to determine if the most extreme values were significant outliers (GraphPad). All means were compared using a protected t-test. Differences were considered significant at P < 0.05 and a trend when P < 0.10. Results are presented as least squared mean (ls means S.E.M.).

## Results

### Prenatal fed restriction did not affect the body weight of F0 females

Similar BW was recorded between control and FR F0 females at the time of AI (3.90 ± 0.12 kg and 4.04 ± 0.08 kg for C and FR groups, respectively) and parturition (4.62 ± 0.12 kg and 4.60 ± 0.08 kg for C and FR groups, respectively), although significant differences were observed from AI to parturition in both experimental groups (P < 0.05).

### Prenatal feed restriction did not impact offspring performance at parturition, weaning and juvenile period

No significant differences were observed between the C and FR groups in the total number of live kits at parturition (11.4 ± 0.7 and 11.1 ± 0.6) and number of live-born kits (10.5 ± 0.8 and 10.7 ± 0.7). However, a trend toward a higher number of stillborns was observed in the C group (0.9 ± 0.3 and 0.3 ± 0.3; P = 0.0542). Litter weight at birth tended to be lower in the C group compared to FR (584.9 ± 21.5 g and 636.3 ± 18.8 g), although this difference was not statistically significant (P = 0.07).

**Fig 3:**
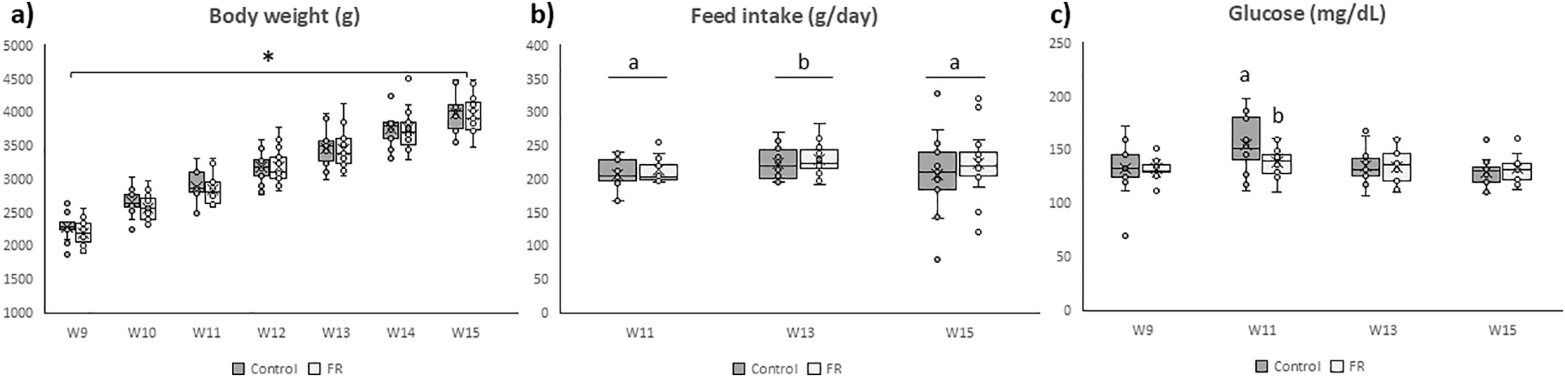
Body weight, feed intake, and blood glucose levels during the juvenile phase of female offspring (F1). Data correspond to daughters of dams fed *ad libitum* (C group, n = 13) or subjected to moderate food restriction during the first two-thirds of gestation (FR group, n = 17). Body weight (a) and feed intake (b) were recorded weekly from 9 to 15 weeks of age, and blood glucose (c) was measured biweekly. Each dot represents one animal; lines indicate least squares means ± SEM. Different superscripts indicate significant differences between groups [^a, b^ (P < 0.05)] or among time points [# (P < 0.0001)].

At weaning, both litter weight (6218.3 ± 195.0 g and 5899.0 ± 166.4 g) and litter size (9.9 ± 0.2 and 9.8 ± 0.1 kits) were comparable between the C and FR groups. From week 9 to week 15 (juvenile phase) the BW of F1 females increased significantly as expected in both groups (P < 0.0001) without significant differences between them (Fig. 3a). Average daily feed intake was comparable between groups (Fig 3b). However, a significant effect of time was detected (P < 0.05), with increased intake observed at week 13 in both groups. The interaction between group and time was not statistically significant.

Glycemic profile did not differ significantly between groups (Fig 3c). Nevertheless, a significant increase in glucose levels was observed in the C group at week 11 compared to FR one (P < 0.001).

### Prenatal fed restriction altered hepatic enzyme levels in F1 females at the onset of their reproductive life, without affecting body composition, or glycemic and lipid profiles

At 16 weeks, females of both experimental groups reached similar mean BW and estimated BC values (Table 2).

**Table 2:** Body weight (BW) and estimated body composition recorded in F1 sexually mature rabbit females (16 weeks old) born to dams subjected to moderate feed restriction (FR group) for 3 weeks or fed *ad libitum* (C group) throughout pregnancy. After birth, all offspring were fed *ad libitum.*

	Control (C)	Feed restriction (FR)
**Number of animals**	n = 13	n = 17
**BW (g)**	4243.46 ± 71.35	4190.71 ± 62.38
**Water (%)**	62.41 ± 3.13	62.46 ± 1.52
**Ash (%)**	3.11 ± 0.10	3.13 ± 0.07
**Protein (%)**	18.16 ± 0.26	18.19 ± 0.16
**Lipids (%)**	12.72 ± 2.91	12.65 ± 1.44
**Energy (kJ/100g)**	1018.22 ± 133.44	1017.03 ± 64.47

Data presented as the mean ±SEM.

No significant effect of maternal FR was observed on serum parameters related to glucose and lipid metabolism. However, hepatic enzyme ALT was significantly higher in F1 females from restricted mothers (P < 0.02). The ratio AST/ALT tended to be higher in FR group (P = 0.09) (Table 3).

**Table 3:** Metabolic serum parameters and hepatic enzyme levels of sexually matured F1 rabbit females (16 weeks old) born to dams subjected to moderate feed restriction (FR group) for 3 weeks or fed *ad libitum* (C group) during pregnancy at ovulation time point.

	Control (C) (n = 13)	Feed restriction (FR) (n = 17)
**Glycemic profile**		
**Glucose (mg/dL)**	128.61 ± 3.85	132.70 ± 3.37
**Insulin (mU/L)**	28.49 ± 4.56	29.93 ± 3.92
**Homa IR**	9.1 ± 1.97	9.1 ± 1.73
**Ratio glucose/insulin**	6.20 ± 1.95	6.29 ± 0.83
**Lipid profile**		
**Triglycerides (mg/dL)**	56.14 ± 5.12	62.58 ± 4.5
**Cholesterol (mg/dL)**	76.60 ± 6.29	69.95 ± 5.45
**Ratio triglycerides/cholesterol**	0.86 ± 0.13	1.05 ± 0.11
**Hepatic profile**		
**ALT (U/L)**	18.2 ± 2.17^a^	25.98 ± 1.91^b^
**AST (U/L)**	23.57 ± 2.65	28.3 ± 2.22
**Ratio AST/ALT**	1.49 ± 0.14*	1.16 ± 0.12*

HOMA IR index: [insulin (mU/L) × (glucose (mg/dL)/18)]/22.5; ALT: serum alanine transaminase; AST: serum aspartate transaminase. Data presented as mean ± sem. Different superscripts indicate significant differences between groups (^ab^ P < 0.02); * (P = 0.09).

### Prenatal feed restriction did not alter AMH levels, ovulation rate, or the overall ovarian steroidogenic response, except for an increased P4/E2 ratio at ovulation in F1 females

Mean serum AMH concentrations were similar in the C and FR groups at 14 h post-GnRH (0.50 ± 0.15 and 0.76 ± 0.53 ng/mL, respectively) and 84 h post-AI (0.54 ± 0.28 and 0.55 ± 0.20 ng/mL, respectively).

**Fig 4:**
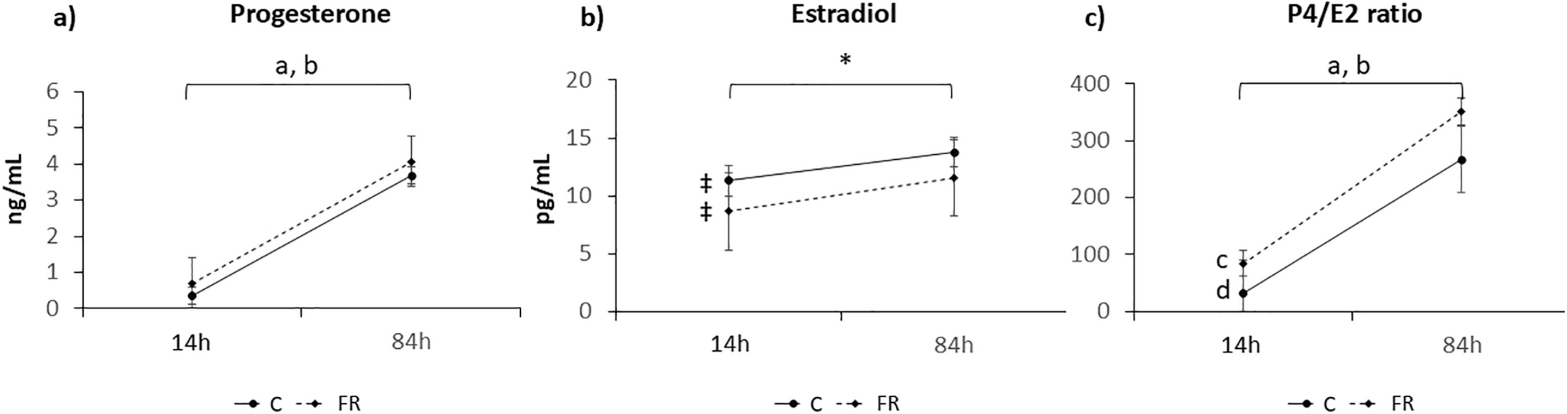
Steroid hormone concentrations in female offspring (F1). Serum levels of (a) progesterone (P4), (b) estradiol (E2), and (c) the progesterone-to-estradiol ratio (P4/E2) were measured in 16-week-old female offspring of dams subjected to moderate food restriction during the first two-thirds of gestation (FR group) or fed ad libitum throughout pregnancy (C group). Number of does: C group, 14 h (n = 6) and 84 h (n = 7); FR group, 14 h (n = 8) and at 84 h (n = 9) after ovulation. Data are presented as mean ± SEM. Different superscripts indicate significant differences [^a,b^ (P < 0.001)] or a trend [(*) P = 0.07] between time points (14 h vs. 84 h after ovulation), and [^c,d^ (P < 0.05)], or a trend [‡ (P = 0.06)] between experimental groups.

Mean number of CL per animal was similar in C and FR (13.00 ± 2.82 and 11.5 ± 3.81, respectively). As depicted in Fig 4a, serum P4 concentrations significantly increased (P < 0.001) from 14 h to 84 h post-GnRH, but no significant differences were observed between the two experimental groups at both time points examined (when COCs or embryos were recovered). E2 tended to be higher in C than FR group (P = 0.06, Fig 4b) at 14 h yet was similar in both groups at 84 h post-GnRH. However, the ratio P4/E2 was significantly higher in FR compared to C (P < 0.05) at 14 h (ovulation time), but no differences were found at the time of embryo recovery (84h) (Fig 4c). In both experimental groups, this ratio increased from 14 h to 84 h after ovulation (P < 0.001).

### Prenatal fed restriction modulates transcript abundance of oxidative stress and apoptosis-related genes in cumulus cells, oocytes and embryos in F1 females

**Fig 5:**
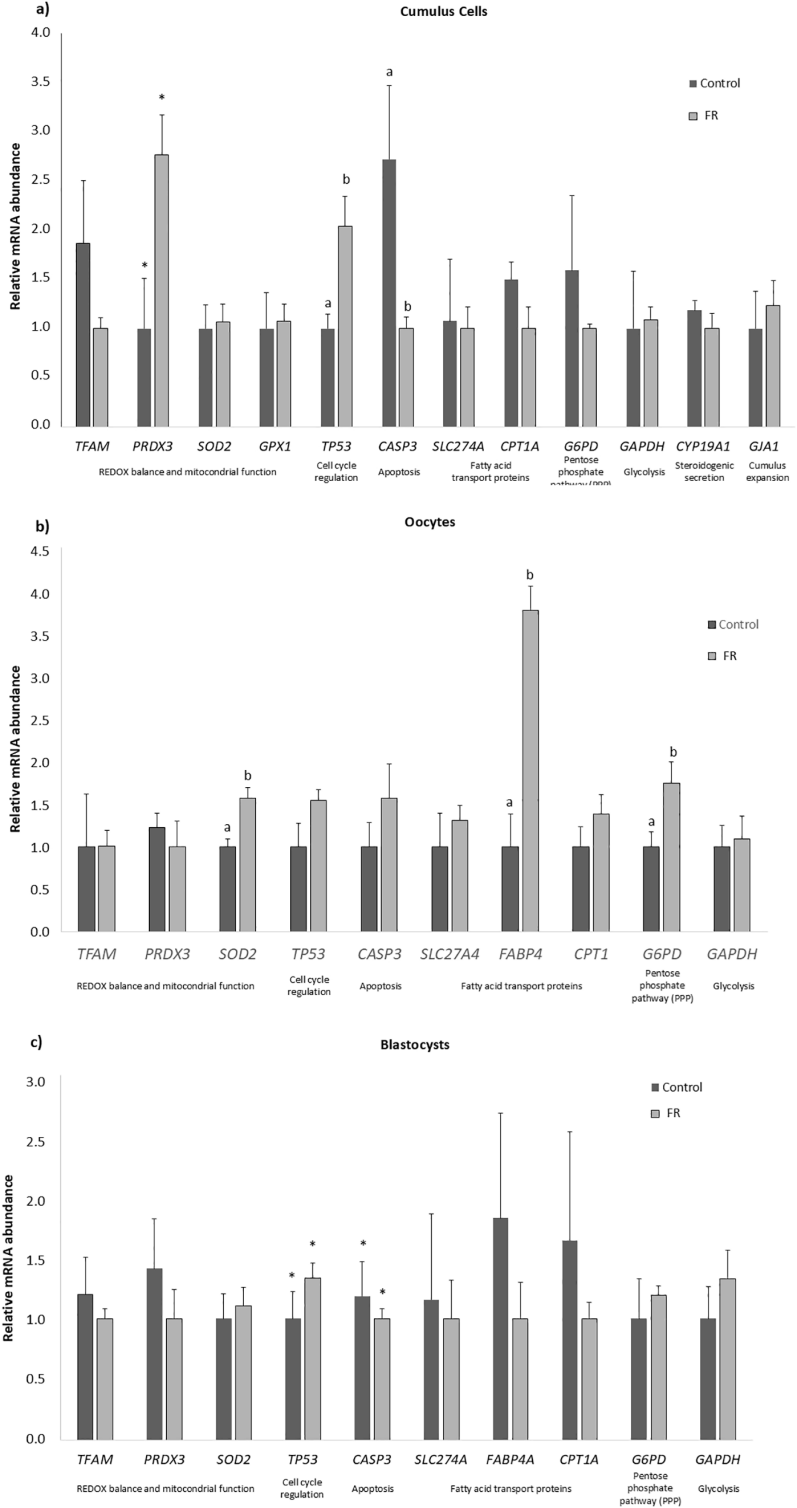
Relative mRNA abundance of target genes in (a) oocytes, (b) cumulus cells (CCs), and (c) blastocysts from female offspring (F1). Samples were obtained from 16-week-old female offspring (F1) of dams subjected to moderate food restriction during the first two-thirds of gestation (FR group) or fed ad libitum throughout pregnancy (C group). Group C: n = 41 oocytes with their corresponding CCs, and 32 blastocysts; Group FR: n = 56 oocytes with their corresponding CCs, and 40 blastocysts. Data are presented as the mean ± SEM. Bars with different superscripts indicate significant differences between C and FR groups for each gen [^a, b^ (P < 0.05)] or a trend [(*) P = 0.07].

Transcript abundances in oocytes, their corresponding CCs, and blastocysts are shown in Figs 5a- 5c, respectively. In oocytes, *SOD2, FABP4,* and *G6PD* poly(A) mRNA expression levels were significantly upregulated in the FR group (P < 0.05). No significant differences were observed in the other transcripts analysed. In CCs, *TP53* mRNA expression was significantly upregulated in FR (P < 0.05), whereas *CASP3* mRNA expression was significantly lower in FR-CCs compared to those from group C (P < 0.05). *PRDX3* mRNA expression tended to be higher in FR compared to C (P = 0.06). No significant differences were observed in the other transcripts analysed. Blastocyst-mRNA abundances of the examined transcripts were similar in the two experimental groups. In blastocysts derived from FR group, *TP53* gene expression tended to be upregulated (P = 0.09), and *CASP3* gene expression downregulated (P = 0.09).

### Prenatal fed restriction improved *in vivo* blastocyst expansion rates and embryo cell count in F1 females

**Fig 6:**
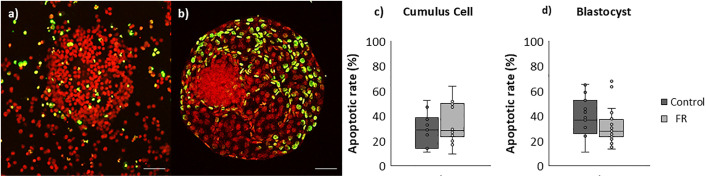
Representative images of apoptosis in cumulus-oocyte complexes (COCs),and expanded blastocysts from female offspring. (a) COCs and (b) expanded blastocysts from F1 females. Apoptosis was evaluated by the TUNEL assay. Confocal images (x40) show maximal projection of a complete COCs (a) and blastocysts (b) illustrating the overlap between nuclei stained with propidium iodide (red) and TUNEL-positive apoptotic cells (green). Graphs (c) and (d) show the apoptotic rate in cumulus cells and blastocysts, respectively, from daughters of dams fed *ad libitum* (C group) or subjected to moderate food restriction during the first two-thirds of gestation (FR group). COCs: C (n = 11), FR (n = 14); embryos: C (n = 16), FR (n = 27). Each dot represents one COC or embryo; lines indicate least squares means ± SEM.

The COC recovery rate at 14 h post-GnRH was comparable between groups (94.87 ± 25.39% and 86.95 ± 24.73%, in group C and FR, respectively). Apoptosis percentages were similar in CCs from C and FR oocytes at ovulation (32.96 ± 14.70% and 28.80 ± 14.30%, respectively) (Fig 6).

**Fig 7:**
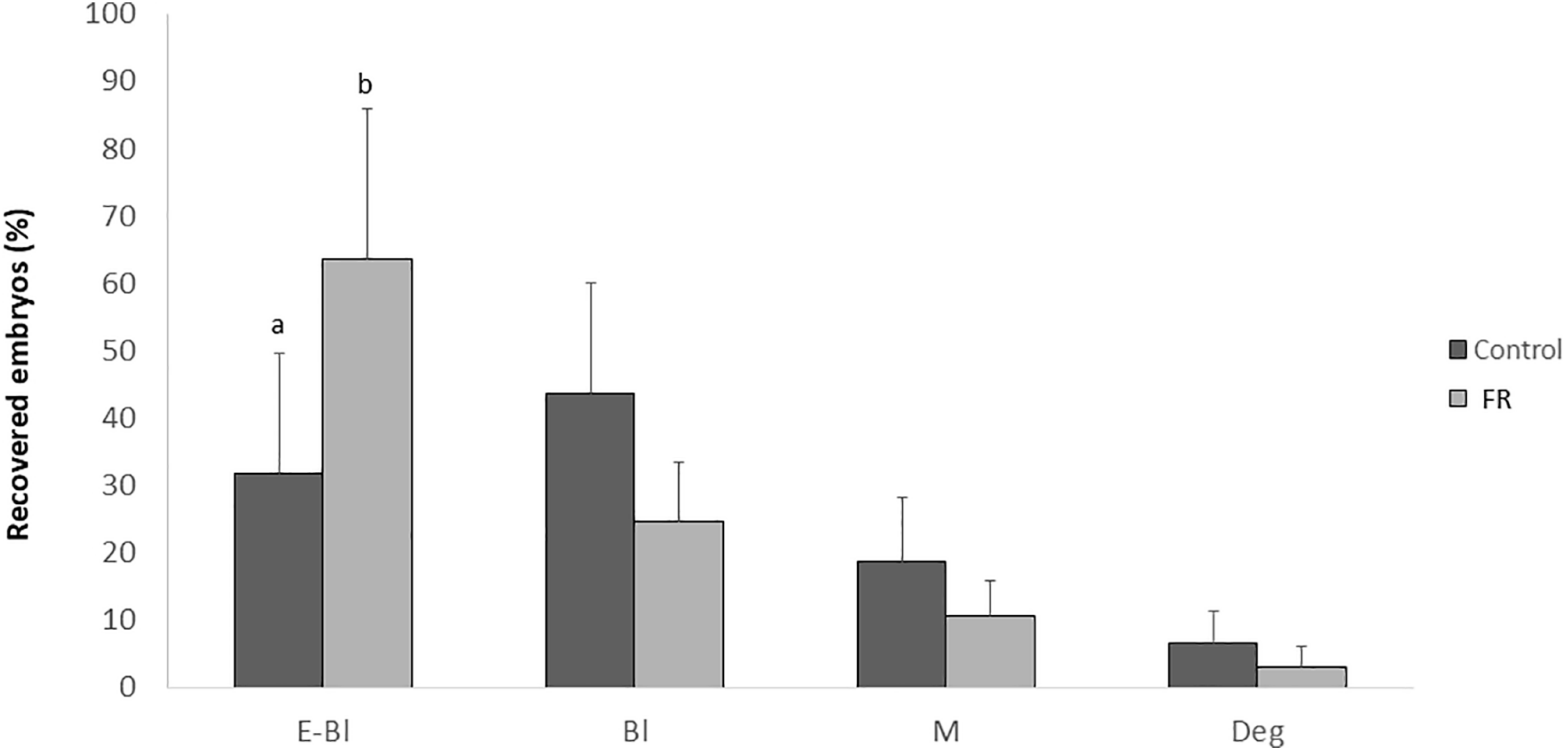
*In vivo* embryo development. Embryos were recovered 84h after GnRH administration and artificial insemination in 16-week-old female offspring (F1) of dams subjected to moderate food restriction during the first two-thirds of gestation (FR, group; n = 9) or fed *ad libitum* throughout pregnancy (C group, n = 7). The total number of embryos analysed was 58 in the C group and 78 in the FR group. Developmental stages were classified as expanded blastocysts (E-Bl), early blastocysts (Bl), morulae (M), or retarded/degenerate embryos (Deg). Data presented as mean ± SEM. Different superscripts indicate significant differences between experimental groups [a, b (P < 0.005)].

The embryo recovery rate did not differ significantly between treatments (81.69 ± 28.62% and 83.87 ± 20.07%, in C and FR, respectively). Likewise, percentages of recovered blastocysts, morulae, unfertilized oocytes, and degenerated embryos were comparable across groups. However, group FR showed a significantly higher number of expanded blastocysts compared to group C (P < 0.005; Fig 7). Embryo cell counts were significantly higher in FR than C group (3218.5 ± 640.28 and 1101.0 ± 270.17 cells, P < 0.05). Apoptosis rate did not significantly vary between the groups (39.49 ± 15.9% and 32.40 ± 17.9%, groups C and FR, respectively) (Fig 6).

**Fig 8:**
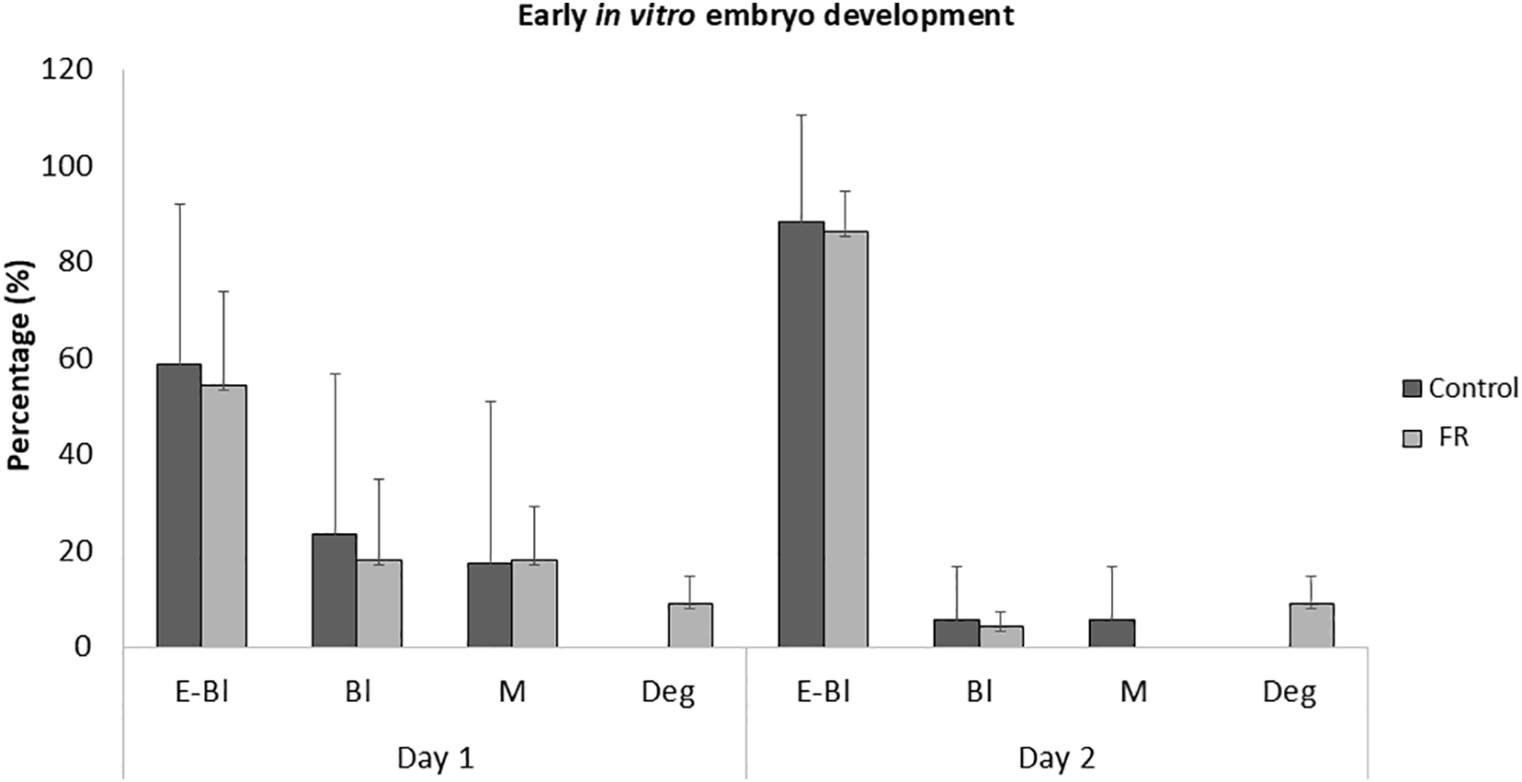
*In vitro* embryo development. Rabbit embryos recovered from 16-week-old female offspring (F1) of dams subjected to moderate food restriction during the first two-thirds of gestation (FR, group) or fed *ad libitum* throughout pregnancy (C group) were cultured *in vitro* for 48h. The number of embryos cultured was 17 in the C group and 22 in the FR group. Developmental stages were classified as morulae (M), early blastocysts (Bl), expanded blastocysts (E-Bl), or degenerated embryos (Deg). Data are presented as mean ± SEM.

As shown in Fig 8, embryo development following 48 h of *in vitro* culture was similar in C and FR groups.

## Discussion

In high-income countries, women frequently adopt dietary restriction strategies to lose weight, which may inadvertently continue into early pregnancy. Such in utero nutritional imbalances -particularly maternal FR- can adversely affect the reproductive potential of the offspring. Emerging evidence highlights the influence of maternal nutrition on oocyte quality in F1 offspring, providing insights into the mechanisms that govern F2 oocyte development and their potential consequences for subsequent reproductive performance. In the present study, animals were subjected to moderate FR during the first two-thirds of gestation, at levels comparable to those observed in both human and animal dietary regimens [63]. In rabbits, this gestational window encompasses critical developmental stages, including preimplantation embryogenesis (0–7 days) and organogenesis (7–18/19 days) [64]. Specifically, first germ cells can be found from day 9 of pregnancy and the gonads are evident from day 14 [50]. Our experimental model demonstrates that moderate maternal FR during this period modulates the expression of genes associated with oxidative stress, apoptosis and fatty acid uptake in oocytes of the F1 female offspring. These findings suggest that FR more than induced adverse effects exerts a potential adaptative response by the activation of compensatory intracellular responses that may support early embryonic development in juvenile females at the onset of their reproductive life.

Although both maternal overnutrition and undernutrition during gestation have been shown to adversely affect offspring growth and metabolic function [65,66], the level of FR applied in this study did not result in in overt detrimental effects on birth weight, survival or postnatal growth. Previous studies have shown that maternal FR during early gestation exerts a limited impact on these parameters in cows [28], although it may still induce metabolic alterations in the offspring [67]. In contrast, nutrient restriction during late gestation has been consistently linked to reduced birth weight in several species, including rats [68], cattle [69,70], and sheep [71]. This increased vulnerability is likely related to the exponential phase of fetal growth during late gestation, when nutrient demands are maximal and sensitivity to dietary restriction perturbations is heightened [72].

In the present study, the restoration of adequate maternal intake during the final third of gestation may may have enabled compensatory fetal growth in the FR group, resulting in birth and weaning outcomes comparable to those of control offspring [42]. In rabbits,moderate and transient FR (30%), during pregnancy has been shown to alter hormonal and metabolic parameters associated with adult disease risk, such as hyperglycaemia, insulin resistance, and increase in thyroxine levels, mirroring alterations described in humans [33]. Notably, when FR is limited to early or mid-gestation, these parameters return to physiological levels by late pregnancy, suggesting the activation of adaptive maternal and fetal responses.

Similarly, studies using a comparable FR model,as applied in the current work, have reported normalized maternal glycaemic and lipid profiles at term, despite the presence of hyperinsulinemia, insulin resistance, and hypertriglyceridemia in near-term fetuses [42]. These fetal metabolic changes may represent early adaptive responses to a nutrient-limited intrauterine environment, although they could also confer increased susceptibility to metabolic disorders such as fatty liver disease, type 2 diabetes, and obesity later in life [9]. In the present study, no significant alterations were observed in growth, feed intake, or glycemic profile of female offspring during juvenile period. Likewise, at the onset of reproductive life, BW, BC, and glycemic and lipid profiles, remained comparable between groups.

These findings are consistent with previous studies in juvenile sheep exposed to maternal undernutrition during early to mid-gestation [73], cows nutritionally restricted during the first trimester [28], mice exposed to a periconceptional protein-restricted environment [9], and rabbits undergoing 50% dietary restriction during gestation [74], all of which described preserved postnatal growth and general health in offspring. Collectively, these data support the notion that adequate postnatal nutrition may mitigate or compensate for prenatal nutritional challenges, thereby facilitating the normalization of key physiological functions [35,43,74,75].

Nevertheless, the detection of elevated serum levels of ALT in F1 female offspring of FR dams suggests the presence of subtle but persistent hepatic alterations [76–79] that were not accompanied by overt growth or metabolic disturbances during early and juvenile life.The prenatal environment also plays a key role in shaping adult ovarian folliculogenesis and steroidogenesis. AMH is widely recognized as a reliable indirect marker of ovarian reserve, as its levels correlate with the number of growing follicles across species, including rodents [80], humans [81], cows [28,82], dogs, guinea pigs, and horses [83]. In rabbits, AMH levels have been shown to correlate particularly well with the number of secondary and antral follicles [84]. In our study, AMH levels were comparable in the offspring of control and fed-restricted mothers, consistent with previous reports [85]. By contrast, studies in other species have demonstrated that maternal FR to 50% during gestation can reduce AMH levels in young adult offspring [30], indicating a compromised ovarian follicular reserve [25–27,37,86]. These interspecies discrepancies may be attributed to differences in the timing of ovarian reserve establishment. Unlike species such as the mice, where primordial follicle formation begins around days 12–13 of gestation, rabbits initiate folliculogenesis exclusively after birth [87]. Thus, maternal FR limited to early and mid-gestation is unlikely to directly impair ovarian reserve formation of F1 female rabbits. In contrast, in species such as sheep and cattle, maternal undernutrition during early gestation has been shown to delay fetal follicle formation and reduce the number of growing follicles in female offspring [24,28], despite minimal effects on prenatal or postnatal growth were evidenced [88]

Our findings also suggest that prenatal FR did not impair the ovarian response to treatment with a short-acting GnRH analogue. The number of COCs retrieved was similar between groups, and serum progesterone (P4) concentrations after ovulation and at the early post-ovulatory window (up to 84 hours) remained within physiological ranges, indicating functional corpora lutea able of supporting early embryo development [89]. Although an early rise in P4 at the time of GnRH administration can potentially shift the endometrial receptivity window and disrupt embryo-maternal synchrony [90,91] (leading to reduced implantation rates and poorer pregnancy outcomes), this effect was not evident in our model. Despite not measuring LH levels directly, the absence of a premature P4 increase 14 hours post-GnRH supports the conclusion that premature luteinization did not occur, consistent with previous observations [92]. Interestingly, an elevated P4/E2 ratio was detected at ovulation in offspring of undernourished mothers. In clinical reproductive contexts, this ratio has been proposed as a more reliable marker of pregnancy outcomes than P4 levels alone [93–95], although the supporting evidence remains inconclusive and thresholds vary depending on the GnRH protocol applied [92,96,97]. Moreover, species-specific differences must be considered, as rabbits are induced ovulators, in contrast to humans, which may limit the extrapolation of these findings. In our study, the elevated P4/E2 ratio in F1 females from FR group did not compromise luteal function nor negatively impact embryonic development. On the contrary, embryos from the FR group exhibited more advanced developmental stages, with a higher proportion reaching the expanded blastocyst stage at the time of recovery, an indicator of greater developmental competence [98]. These findings suggest the possible involvement of adaptive or compensatory mechanisms, either intrinsic to the oocyte or mediated by the oviductal microenvironment, that may confer a developmental advantage. Nevertheless, in the absence of significant differences in gene expression profiles and given the similar *in vitro* development observed under standardized culture conditions, the underlying mechanisms remain unclear and warrant further investigation.

Although less investigated, maternal undernutrition has also been linked to altered expression of key ovarian steroidogenic enzymes, such as P450SCC and aromatase, which are essential for sex steroid synthesis in the offspring ovary [99–101]. In our study, although serum E2 levels were slightly higher in the control group, no significant differences were observed, neither in hormone concentrations nor in *CYP19A1* expression in CCs. These results are consistent with those of Maia et al. [35] who reported no significant changes in serum P4 or E2 concentrations following in utero nutritional restriction in cattle. Conversely, other studies have reported disrupted ovarian steroidogenesis [29], reduced CL number [24,30], and decreased plasma P4 levels in adult F1 females [2,102]. Collectively, these outcomes may reflect differences in the severity, duration, and type of maternal nutritional restriction, particularly in models involving protein deprivation rather than global caloric restriction [26,30,37].

At the molecular level, maternal FR induced transcriptional changes in genes associated with cell cycle regulation, apoptosis, and oxidative stress within the COCs of female offspring. TP53, often referred to as “the guardian of the genome,” orchestrates cell cycle arrest and apoptosis via mitochondrial caspases. It regulates an appropriate cellular response to various stress signals [103]. Specifically, TP53 activation in preovulatory mouse granulose cells impairs ovulation and fertilization [104] and promotes apoptosis in macaque granulosa cells [105]. In the present study, *TP53* was upregulated in CCs from FR group, suggesting a potential response to prenatal stress signals. Notably, this increase was accompanied by downregulation of its effector, *CASP3*which may indicate a protective or compensatory adaptation rather than the activation of apoptotic pathways [106]. Together with the similar expression genes related to CCs expansion (*CJA1*), steroidogenic activity (*CYP19A1*), ovulation rate and apoptotic rate determined using the TUNEL assay in both groups, these findings suggest that maternal FR did not exert a detrimental effect on F2-COCs functionality. This observation aligns with earlier findings showing increased *TP53* and *CASP3* expression in *in vitro* matured oocytes, reflecting enhanced DNA repair and apoptotic regulation compared to *in vivo* matured COCs [52]. Moreover, the ancestral function of TP53 is linked to ensure fecundity and embryo viability rather than tumour suppression [103], supporting the involvement in the acquisition of developmental competence [107] and the selective elimination of damaged oocytes and embryos [108], instead of acting merely as an inductor of embryo loss [109,110].

Oxidative stress is a recognized mediator of long-term physiological changes induced by early-life nutritional insults [92,108,109]. In oocytes, SOD2 is involved in oxidative responses and mitochondrial activity, as it encodes a mitochondrial protein that catalyses the dismutation of superoxide into H_2_O_2_. Its upregulation in oocytes from the FR group indicates activation of redox homeostasis mechanisms, potentially contributing to the preservation of mitochondrial function and biogenesis. The unchanged expression of *PRDX3* and *TFAM* mRNAs in both groups further supports this notion. Notably, mRNA levels of *TP53* and *CASP3* in oocytes were also similar between groups, indicating a balance between oxidative stress management and apoptotic signalling, in agreement with previous findings in rabbit oocytes [52]. PRDX3 catalyses the reduction of mitochondrial H₂O₂ and it is considered a marker of mitochondrial oxidative stress and apoptosis [110,111]. In our study, the slightly increased expression of *PRDX3* in CCs from the FR group may reflect the activation of antioxidant defence. *PRDX3* upregulation has previously been associated with increased mitochondrial DNA copy number, enhanced CCs survival, and improved oocyte developmental competence to the blastocyst stage in gilts [112].

Additionally, *G6PD* expression was upregulated in oocytes from the FR group. G6PD is a cytosolic enzyme that catalyzes the first rate-limiting step of the pentose phosphate pathway (PPP), which primarily generates NADP, a key electron donor for antioxidant defence and reductive biosynthetic reactions. Increased *G6PD* expression may therefore enhanced protection against oxidative damage, supporting meiotic progression and developmental potential to reach the blastocyst stage [113]. Overall, these data suggest that the antioxidant defence mechanisms in oocytes of F1 females remain functionally intact under prenatal FR conditions. Nevertheless, findings regarding G6PD expression and oocyte competence appears to be species-specific, as controversial results have been reported in rats [113], sheep [114], rabbits [52], and cattle [115]. Finally, fatty acid binding protein 4 (FABP4), a cytoplasmic protein involved in fatty acid uptake, transport, and metabolism was upregulated in oocytes from the FR group. FABP4 is found in adipocytes and granulosa cells during follicular atresia in lambs [102] and mice [116]. Its upregulation may reflect prenatal metabolic programming of lipid homeostasis. While such adaptations could be under conditions of limited nutrient availability, they may also predispose offspring to metabolic disorders later in life when nutrient supply is restored (as in the present model), potentially even affecting the F2 generation. To our knowledge, this is the first report describing altered *FABP4* expression in oocytes in the context of prenatal undernutrition therefore the interpretation of this result requires prudence. The limited sample size of F1 females that achieved the onset of reproductive life and the focus on a selected set of candidate genes, although biologically relevant, do not capture the full spectrum of molecular responses to maternal undernutrition. Further studies with larger cohorts and functional analyses beyond gene expression are needed to elucidate how these genes influence reproductive outcomes and health in following generation.

## Conclusions

Our results indicate that the reproductive phenotype of female offspring exposed to maternal FR during first two thirds of gestation may be modulated through changes in oocyte gene expression, particularly in pathways related to oxidative stress, apoptosis, and lipid metabolism. Importantly, the observed molecular changes are more consistent with manifestations of developmental plasticity than with impairment *per se*, and appear to function predominantly as compensatory mechanisms, that contribute to the preservation of oocyte competence and early embryo development in F1 females exposed to suboptimal prenatal conditions. Nevertheless, these findings should be interpreted cautiously, as the specific causal mechanism remain to be elucidated. Re-feeding at the end of pregnancy after maternal FR appears to mitigate long-term adverse effects on offspring health indicators such as growth, body composition and glycaemic and lipid metabolism but, the detection of liver damage biomarkers suggests subtle physiological impacts. Overall, these results underscore the intrinsic plasticity of female offspring to prenatal nutritional challenges and highlight the need for further research to elucidate how early-life molecular programming influences fertility and health outcomes in subsequent generations.

## Supporting information

S1 Data Raw data 1. (XLSX)

S2 Data Raw data 2. (XLSX)

## References

[1] Barker DJP. The origins of the developmental origins theory. J Intern Med. 2007;261(5):412–7. doi: 10.1111/j.1365-2796.2007.01809.x 17444880

[2] Sloboda DM, Howie GJ, Pleasants A, Gluckman PD, Vickers MH. Pre- and postnatal nutritional histories influence reproductive maturation and ovarian function in the rat. PLoS One. 2009;4(8):e6744. doi: 10.1371/journal.pone.0006744 19707592 PMC2727050

[3] Godfrey KM, Gluckman PD, Hanson MA. Developmental origins of metabolic disease: life course and intergenerational perspectives. Trends Endocrinol Metab. 2010;21(4):199–205. doi: 10.1016/j.tem.2009.12.008 20080045

[4] Gluckman PD, Hanson MA, Cooper C, Thornburg KL. Effect of in utero and early-life conditions on adult health and disease. N Engl J Med. 2008;359(1):61–73. doi: 10.1056/NEJMra0708473 18596274 PMC3923653

[5] Gluckman PD, Hanson MA, Pinal C. The developmental origins of adult disease. Matern Child Nutr. 2005;1(3):130–41. doi: 10.1111/j.1740-8709.2005.00020.x 16881892 PMC6860944

[6] Roseboom T, de Rooij S, Painter R. The Dutch famine and its long-term consequences for adult health. Early Hum Dev. 2006;82(8):485–91. doi: 10.1016/j.earlhumdev.2006.07.001 16876341

[7] Tobi EW, Slieker RC, Luijk R, Dekkers KF, Stein AD, Xu KM, et al. DNA methylation as a mediator of the association between prenatal adversity and risk factors for metabolic disease in adulthood. Sci Adv. 2018;4:eaao4364. doi: 10.1126/sciadv.aao4364 PMC5792223 29399631

[8] Neel JV. Diabetes mellitus: a “thrifty” genotype rendered detrimental by “progress”?. Am J Hum Genet. 1962;14(4):353–62. 13937884 PMC1932342

[9] Fleming TP, Watkins AJ, Velazquez MA, Mathers JC, Prentice AM, Stephenson J, et al. Origins of lifetime health around the time of conception: causes and consequences. Lancet. 2018;391(10132):1842–52. doi: 10.1016/S0140-6736(18)30312-X 29673874 PMC5975952

[10] Yao S, Lopez-Tello J, Sferruzzi-Perri AN. Developmental programming of the female reproductive system-a review. Biol Reprod. 2021;104(4):745–70. doi: 10.1093/biolre/ioaa232 33354727

[11] Roseboom TJ, van der Meulen JH, Ravelli AC, Osmond C, Barker DJ, Bleker OP. Effects of prenatal exposure to the Dutch famine on adult disease in later life: an overview. Mol Cell Endocrinol. 2001;185(1–2):93–8. doi: 10.1016/s0303-7207(01)00721-3 11738798

[12] Global Nutrition Report. 2022 Global Nutrition Report: Executive Summary. [Internet]. 2022 [cited 2025 May 28]. Available from: https://globalnutritionreport.org/reports/2022-global-nutrition-report/executive-summary/

[13] World Health Organization. Fact Sheet: Malnutrition. [Internet]. 2021 [cited 2025 May 28]. Available from: https://www.who.int/news-room/fact-sheets/detail/malnutrition

[14] Crozier SR, Robinson SM, Godfrey KM, Cooper C, Inskip HM. Women’s dietary patterns change little from before to during pregnancy. J Nutr. 2009;139(10):1956–63. doi: 10.3945/jn.109.109579 19710161 PMC3113465

[15] Andrés S, Madsen O, Montero O, Martín A, Giráldez FJ. The Role of Feed Restriction on DNA Methylation, Feed Efficiency, Metabolome, Biochemical Profile, and Progesterone Patterns in the Female Filial Generation (F1) Obtained From Early Feed Restricted Ewes (F0). Front Physiol. 2021;12:779054. doi: 10.3389/fphys.2021.779054 35024036 PMC8745145

[16] Gu L, Liu H, Gu X, Boots C, Moley KH, Wang Q. Metabolic control of oocyte development: linking maternal nutrition and reproductive outcomes. Cell Mol Life Sci. 2015;72(2):251–71. doi: 10.1007/s00018-014-1739-4 25280482 PMC4389777

[17] Tang S-B, Zhang T-T, Yin S, Shen W, Luo S-M, Zhao Y, et al. Inheritance of perturbed methylation and metabolism caused by uterine malnutrition via oocytes. BMC Biol. 2023;21(1):43. doi: 10.1186/s12915-023-01545-x 36829148 PMC9960220

[18] Heard E, Martienssen RA. Transgenerational epigenetic inheritance: myths and mechanisms. Cell. 2014;157(1):95–109. doi: 10.1016/j.cell.2014.02.045 24679529 PMC4020004

[19] Peral-Sanchez I, Hojeij B, Ojeda DA, Steegers-Theunissen RPM, Willaime-Morawek S. Epigenetics in the Uterine Environment: How Maternal Diet and ART May Influence the Epigenome in the Offspring with Long-Term Health Consequences. Genes (Basel). 2021;13(1):31. doi: 10.3390/genes13010031 35052371 PMC8774448

[20] Morgan HD, Sutherland HG, Martin DI, Whitelaw E. Epigenetic inheritance at the agouti locus in the mouse. Nat Genet. 1999;23(3):314–8. doi: 10.1038/15490 10545949

[21] García-García RM, Rebollar PG, Arias-Álvarez M, Sakr OG, Bermejo-Álvarez P, Brecchia G, et al. Acute fasting before conception affects metabolic and endocrine status without impacting follicle and oocyte development and embryo gene expression in the rabbit. Reprod Fertil Dev. 2011;23(6):759–68. doi: 10.1071/RD10298 21791177

[22] Mishina T, Tabata N, Hayashi T, Yoshimura M, Umeda M, Mori M, et al. Single-oocyte transcriptome analysis reveals aging-associated effects influenced by life stage and calorie restriction. Aging Cell. 2021;20(8):e13428. doi: 10.1111/acel.13428 34245092 PMC8373347

[23] Naturil-Alfonso C, Peñaranda DS, Vicente JS, Marco-Jiménez F. Feed restriction regime in a rabbit line selected for growth rate alters oocyte maturation manifested by alteration in MSY2 gene expression. Reprod Domest Anim. 2017;52(6):976–84. doi: 10.1111/rda.13006 28627068

[24] Rae MT, Kyle CE, Miller DW, Hammond AJ, Brooks AN, Rhind SM. The effects of undernutrition, in utero, on reproductive function in adult male and female sheep. Anim Reprod Sci. 2002;72(1–2):63–71. doi: 10.1016/s0378-4320(02)00068-4 12106966

[25] Bernal AB, Vickers MH, Hampton MB, Poynton RA, Sloboda DM. Maternal undernutrition significantly impacts ovarian follicle number and increases ovarian oxidative stress in adult rat offspring. PLoS One. 2010;5(12):e15558. doi: 10.1371/journal.pone.0015558 21179452 PMC3001490

[26] Chan KA, Bernal AB, Vickers MH, Gohir W, Petrik JJ, Sloboda DM. Early life exposure to undernutrition induces ER stress, apoptosis, and reduced vascularization in ovaries of adult rat offspring. Biol Reprod. 2015;92(4):110. doi: 10.1095/biolreprod.114.124149 25810471 PMC4643955

[27] Harrath AH, Alrezaki A, Mansour L, Alwasel SH, Palomba S. Food restriction during pregnancy and female offspring fertility: adverse effects of reprogrammed reproductive lifespan. J Ovarian Res. 2017;10(1):77. doi: 10.1186/s13048-017-0372-x 29282125 PMC5745764

[28] Mossa F, Carter F, Walsh SW, Kenny DA, Smith GW, Ireland JLH, et al. Maternal undernutrition in cows impairs ovarian and cardiovascular systems in their offspring. Biol Reprod. 2013;88(4):92. doi: 10.1095/biolreprod.112.107235 23426432

[29] Harrath AH, Alrezaki A, Alwasel SH, Semlali A. Intergenerational response of steroidogenesis-related genes to maternal malnutrition. J Dev Orig Health Dis. 2019;10(5):587–94. doi: 10.1017/S2040174419000060 30789120

[30] Chan KA, Jazwiec PA, Gohir W, Petrik JJ, Sloboda DM. Maternal nutrient restriction impairs young adult offspring ovarian signaling resulting in reproductive dysfunction and follicle loss. Biol Reprod. 2018;98(5):664–82. doi: 10.1093/biolre/ioy008 29351580

[31] Akbarinejad V, Cushman RA. Developmental programming of reproduction and production in the offspring. Anim Reprod Sci. 2024;267:107520. doi: 10.1016/j.anireprosci.2024.107520 38834404

[32] Ellis-Hutchings RG, Zucker RM, Grey BE, Norwood J Jr, Richards JH, Lau C, et al. Altered health outcomes in adult offspring of Sprague Dawley and Wistar rats undernourished during early or late pregnancy. Birth Defects Res B Dev Reprod Toxicol. 2010;89(5):396–407. doi: 10.1002/bdrb.20265 20973054

[33] Menchetti L, Brecchia G, Canali C, Cardinali R, Polisca A, Zerani M, et al. Food restriction during pregnancy in rabbits: effects on hormones and metabolites involved in energy homeostasis and metabolic programming. Res Vet Sci. 2015;98:7–12. doi: 10.1016/j.rvsc.2014.11.017 25499747

[34] Ashworth CJ, Toma LM, Hunter MG. Nutritional effects on oocyte and embryo development in mammals: implications for reproductive efficiency and environmental sustainability. Philos Trans R Soc Lond B Biol Sci. 2009;364(1534):3351–61. doi: 10.1098/rstb.2009.0184 19833647 PMC2781853

[35] Maia TS, Guimarães HR, Garza V, Pohler KG, Cardoso RC, Williams GL. Early juvenile but not mid-to-late prenatal nutrition controls puberty in heifers but neither impact adult reproductive function†. Biol Reprod. 2022;107(4):1035–45. doi: 10.1093/biolre/ioac123 35703941

[36] Williams GL, Zhang Y, O’Neil MM, Maia TS, West SM, Alves BRC, et al. Interaction of pre- and postnatal nutrition on expression of leptin receptor variants and transporter molecules, leptin transport, and functional response to leptin in heifers†. Biol Reprod. 2023;109(6):892–903. doi: 10.1093/biolre/ioad118 37698264

[37] Winship AL, Gazzard SE, Cullen-McEwen LA, Bertram JF, Hutt KJ. Maternal low-protein diet programmes low ovarian reserve in offspring. Reproduction. 2018;156(4):299–311. doi: 10.1530/REP-18-0247 30306601

[38] Igosheva N, Abramov AY, Poston L, Eckert JJ, Fleming TP, Duchen MR, et al. Maternal diet-induced obesity alters mitochondrial activity and redox status in mouse oocytes and zygotes. PLoS One. 2010;5(4):e10074. doi: 10.1371/journal.pone.0010074 20404917 PMC2852405

[39] Kitagawa Y, Suzuki K, Yoneda A, Watanabe T. Effects of oxygen concentration and antioxidants on the in vitro developmental ability, production of reactive oxygen species (ROS), and DNA fragmentation in porcine embryos. Theriogenology. 2004;62(7):1186–97. doi: 10.1016/j.theriogenology.2004.01.011 15325546

[40] Guérin P, El Mouatassim S, Ménézo Y. Oxidative stress and protection against reactive oxygen species in the pre-implantation embryo and its surroundings. Hum Reprod Update. 2001;7(2):175–89. doi: 10.1093/humupd/7.2.175 11284661

[41] Lopez-Tello J, Arias-Alvarez M, Jimenez-Martinez MA, Garcia-Garcia RM, Rodriguez M, Lorenzo Gonzalez PL, et al. Competition for Materno-Fetal Resource Partitioning in a Rabbit Model of Undernourished Pregnancy. PLoS One. 2017;12(1):e0169194. doi: 10.1371/journal.pone.0169194 28046002 PMC5207739

[42] Garcia-Garcia RM, Arias-Alvarez M, Millan P, Rodriguez Francisco M, Sanchez Rodriguez A, Lorenzo PL, et al. Gestation Food Restriction and Refeeding Compensate Maternal Energy Status and Alleviate Metabolic Consequences in Juvenile Offspring in a Rabbit Model. Nutrients. 2021;13(2):310. doi: 10.3390/nu13020310 33499108 PMC7912334

[43] García-García RM, Arias-Álvarez M, Rodríguez M, Sánchez-Rodríguez A, Formoso-Rafferty N, Lorenzo PL, et al. Effects of feed restriction during pregnancy on maternal reproductive outcome, foetal hepatic IGF gene expression and offspring performance in the rabbit. Animal. 2021;15(11):100382. doi: 10.1016/j.animal.2021.100382 34653786

[44] Fernández-Pacheco C, Millán P, Rodríguez M, Formoso-Rafferty N, Sánchez-Rodríguez A, Lorenzo PL, et al. Influence of Different Regimes of Moderate Maternal Feed Restriction during Pregnancy of Primiparous Rabbit Does on Long-Term Metabolic Energy Homeostasis, Productive Performance and Welfare. Animals (Basel). 2021;11(9):2736. doi: 10.3390/ani11092736 34573702 PMC8470312

[45] Ogonuki N, Inoue K, Miki H, Mochida K, Hatori M, Okada H, et al. Differential development of rabbit embryos following microinsemination with sperm and spermatids. Mol Reprod Dev. 2005;72(3):411–7. doi: 10.1002/mrd.20363 16078271

[46] Fan J, Watanabe T. Transgenic rabbits as therapeutic protein bioreactors and human disease models. Pharmacol Ther. 2003;99(3):261–82. doi: 10.1016/s0163-7258(03)00069-x 12951161

[47] Fan J, Kitajima S, Watanabe T, Xu J, Zhang J, Liu E, et al. Rabbit models for the study of human atherosclerosis: from pathophysiological mechanisms to translational medicine. Pharmacol Ther. 2015;146:104–19. doi: 10.1016/j.pharmthera.2014.09.009 25277507 PMC4304984

[48] Graur D, Duret L, Gouy M. Phylogenetic position of the order Lagomorpha (rabbits, hares and allies). Nature. 1996;379(6563):333–5. doi: 10.1038/379333a0 8552186

[49] Fischer B, Chavatte-Palmer P, Viebahn C, Navarrete Santos A, Duranthon V. Rabbit as a reproductive model for human health. Reproduction. 2012;144(1):1–10. doi: 10.1530/REP-12-0091 22580370

[50] Lopez-Tello J, Arias-Alvarez M, Gonzalez-Bulnes A, Sferuzzi-Perri AN. Models of Intrauterine growth restriction and fetal programming in rabbits. Mol Reprod Dev. 2019;86(12):1781–809. doi: 10.1002/mrd.23271 31538701

[51] Blas C de, Mateos GG. Feed formulation. Nutrition of the rabbit. CABI. 2010. p. 222–32. doi: 10.1079/9781845936693.0222

[52] Arias-Álvarez M, García-García RM, López-Tello J, Rebollar PG, Gutiérrez-Adán A, Lorenzo PL. In vivo and in vitro maturation of rabbit oocytes differently affects the gene expression profile, mitochondrial distribution, apoptosis and early embryo development. Reprod Fertil Dev. 2017;29(9):1667–79. doi: 10.1071/RD15553 27678473

[53] Arias-Álvarez M, García-García RM, Lorenzo PL, Gutiérrez-Adán A, Sakr OG, González-Bulnes A, et al. Embryo gene expression in response to maternal supplementation with glycogenic precursors in the rabbit. Anim Reprod Sci. 2013;142(3–4):173–82. doi: 10.1016/j.anireprosci.2013.10.001 24358512

[54] Arias-Alvarez M, García-García RM, Torres-Rovira L, González-Bulnes A, Rebollar PG, Lorenzo PL. Influence of leptin on in vitro maturation and steroidogenic secretion of cumulus-oocyte complexes through JAK2/STAT3 and MEK 1/2 pathways in the rabbit model. Reproduction. 2010;139(3):523–32. doi: 10.1530/REP-09-0309 20032210

[55] Arias-Alvarez M, García-García RM, Rebollar PG, Revuelta L, Millán P, Lorenzo PL. Influence of metabolic status on oocyte quality and follicular characteristics at different postpartum periods in primiparous rabbit does. Theriogenology. 2009;72(5):612–23. doi: 10.1016/j.theriogenology.2009.04.017 19523677

[56] Pereda N. Evaluación de la técnica del análisis de impedancia bioeléctrica para predecir la composición corporal: aplicación en conejas sometidas a diferentes sistemas de alimentación durante la recría. Madrid: Universidad Politécnica de Madrid. 2010.

[57] Arias-Alvarez M, García-García RM, Rebollar PG, Gutiérrez-Adán A, López-Béjar M, Lorenzo PL. Ovarian response and embryo gene expression patterns after nonsuperovulatory gonadotropin stimulation in primiparous rabbits does. Theriogenology. 2013;79(2):323–30. doi: 10.1016/j.theriogenology.2012.09.019 23154142

[58] Bermejo-Alvarez P, Lonergan P, Rizos D, Gutiérrez-Adan A. Low oxygen tension during IVM improves bovine oocyte competence and enhances anaerobic glycolysis. Reprod Biomed Online. 2010;20(3):341–9. doi: 10.1016/j.rbmo.2009.12.006 20093090

[59] Arias-Álvarez M, García-García RM, López-Tello J, Rebollar PG, Gutiérrez-Adán A, Lorenzo PL. α-Tocopherol modifies the expression of genes related to oxidative stress and apoptosis during in vitro maturation and enhances the developmental competence of rabbit oocytes. Reprod Fertil Dev. 2018;30(12):1728–38. doi: 10.1071/RD17525 29966585

[60] Arias-Alvarez M, Bermejo-Alvarez P, Gutierrez-Adan A, Rizos D, Lorenzo PL, Lonergan P. Effect of leptin supplementation during in vitro oocyte maturation and embryo culture on bovine embryo development and gene expression patterns. Theriogenology. 2011;75(5):887–96. doi: 10.1016/j.theriogenology.2010.10.031 21196029

[61] Schmittgen TD, Livak KJ. Analyzing real-time PCR data by the comparative C(T) method. Nat Protoc. 2008;3(6):1101–8. doi: 10.1038/nprot.2008.73 18546601

[62] Bó GA, Mapletoft RJ. Evaluation and classification of bovine embryos. Anim Reprod. 2013;10(3):344–8.

[63] Zócalo Y, Ungerfeld R, Pérez-Clariget R, Bia D. Maternal nutritional restriction during gestation impacts differently on offspring muscular and elastic arteries and is associated with increased carotid resistance and ventricular afterload in maturity. did not differ significantly between treatmentsAnderson J, Henck J. Fetal development. In: Manning PJ, Ringler DH, Newcomer CE, editors. The Biology of the Laboratory Rabbit. Cambridge, MA: Academic Press; 1994. p. 457.

[64] Reynolds LP, Borowicz PP, Caton JS, Crouse MS, Dahlen CR, Ward AK. Developmental Programming of Fetal Growth and Development. Vet Clin North Am Food Anim Pract. 2019;35(2):229–47. doi: 10.1016/j.cvfa.2019.02.006 31103178

[65] Gardner DS, Ozanne SE, Sinclair KD. Effect of the early-life nutritional environment on fecundity and fertility of mammals. Philos Trans R Soc Lond B Biol Sci. 2009;364(1534):3419–27. doi: 10.1098/rstb.2009.0121 19833652 PMC2781843

[66] Ford SP, Hess BW, Schwope MM, Nijland MJ, Gilbert JS, Vonnahme KA, et al. Maternal undernutrition during early to mid-gestation in the ewe results in altered growth, adiposity, and glucose tolerance in male offspring. J Anim Sci. 2007;85(5):1285–94. doi: 10.2527/jas.2005-624 17224460

[67] Richter HG, Hansell JA, Raut S, Giussani DA. Melatonin improves placental efficiency and birth weight and increases the placental expression of antioxidant enzymes in undernourished pregnancy. J Pineal Res. 2009;46(4):357–64. doi: 10.1111/j.1600-079X.2009.00671.x 19552758

[68] Freetly HC, Ferrell CL, Jenkins TG. Nutritionally altering weight gain patterns of pregnant heifers and young cows changes the time that feed resources are offered without any differences in production. J Anim Sci. 2005;83(4):916–26. doi: 10.2527/2005.834916x 15753348

[69] Niederecker KN, Larson JM, Kallenbach RL, Meyer AM. Effects of feeding stockpiled tall fescue versus summer-baled tall fescue-based hay to late gestation beef cows: I. Cow performance, maternal metabolic status, and fetal growth. J Anim Sci. 2018;96(11):4618–32. doi: 10.1093/jas/sky341 30137366 PMC6247859

[70] Borwick SC, Rae MT, Brooks J, McNeilly AS, Racey PA, Rhind SM. Undernutrition of ewe lambs in utero and in early post-natal life does not affect hypothalamic-pituitary function in adulthood. Anim Reprod Sci. 2003;77(1–2):61–70. doi: 10.1016/s0378-4320(02)00261-0 12654528

[71] Li X, Yan Q, Tang S, Tan Z, Fitzsimmons CJ, Yi K. Effects of maternal feed intake restriction during pregnancy on the expression of growth regulation, imprinting and epigenetic transcription-related genes in foetal goats. Anim Reprod Sci. 2018;198:90–8. doi: 10.1016/j.anireprosci.2018.09.005 30213570

[72] Hyatt MA, Walker DA, Stephenson T, Symonds ME. Ontogeny and nutritional manipulation of the hepatic prolactin-growth hormone-insulin-like growth factor axis in the ovine fetus and in neonate and juvenile sheep. Proc Nutr Soc. 2004;63(1):127–35. doi: 10.1079/PNS2003324 15070443

[73] Symeon GK, Goliomytis M, Bizelis I, Papadomichelakis G, Pagonopoulou O, Abas Z, et al. Effects of gestational maternal undernutrition on growth, carcass composition and meat quality of rabbit offspring. PLoS One. 2015;10(2):e0118259. doi: 10.1371/journal.pone.0118259 25671602 PMC4324905

[74] Wallace J, Bourke D, Da Silva P, Aitken R. Nutrient partitioning during adolescent pregnancy. Reproduction. 2001;122(3):347–57. doi: 10.1530/rep.0.1220347 11597301

[75] Amacher DE. A toxicologist’s guide to biomarkers of hepatic response. Hum Exp Toxicol. 2002;21(5):253–62. doi: 10.1191/0960327102ht247oa 12141396

[76] Ozer J, Ratner M, Shaw M, Bailey W, Schomaker S. The current state of serum biomarkers of hepatotoxicity. Toxicology. 2008;245(3):194–205. doi: 10.1016/j.tox.2007.11.021 18291570

[77] Wang Y, Xiong B, Liang B, Zhao H, Li H, Qian J, et al. Hepatic parenchymal changes following transcatheter embolization and chemoembolization in a rabbit tumor model. PLoS One. 2013;8(8):e70757. doi: 10.1371/journal.pone.0070757 23967098 PMC3743795

[78] Adam GO, Kim G-B, Lee S-J, Lee H-R, Kim S-J, Kang H-S, et al. Long-term oral intake of Panax ginseng improves hypomagnesemia, hyperlactatemia, base deficit, and metabolic acidosis in an alloxan-induced rabbit model. Iran J Basic Med Sci. 2019;22(6):703–9. doi: 10.22038/ijbms.2019.33223.7936 31231500 PMC6570753

[79] Weenen C, Laven JSE, Von Bergh ARM, Cranfield M, Groome NP, Visser JA, et al. Anti-Müllerian hormone expression pattern in the human ovary: potential implications for initial and cyclic follicle recruitment. Mol Hum Reprod. 2004;10(2):77–83. doi: 10.1093/molehr/gah015 14742691

[80] Visser JA, Schipper I, Laven JSE, Themmen APN. Anti-Müllerian hormone: an ovarian reserve marker in primary ovarian insufficiency. Nat Rev Endocrinol. 2012;8(6):331–41. doi: 10.1038/nrendo.2011.224 22231848

[81] Arouche N, Picard J-Y, Monniaux D, Jamin SP, Vigier B, Josso N, et al. The BOC ELISA, a ruminant-specific AMH immunoassay, improves the determination of plasma AMH concentration and its correlation with embryo production in cattle. Theriogenology. 2015;84(8):1397–404. doi: 10.1016/j.theriogenology.2015.07.026 26298408

[82] van Rooij IAJ, Broekmans FJM, te Velde ER, Fauser BCJM, Bancsi LFJMM, de Jong FH, et al. Serum anti-Müllerian hormone levels: a novel measure of ovarian reserve. Hum Reprod. 2002;17(12):3065–71. doi: 10.1093/humrep/17.12.3065 12456604

[83] Böhmer F, Erber K, Ewringmann A, Klein R, Reese S, Böhmer C, et al. Anti-Müllerian hormone concentrations in female rabbits and its relation to spay status, pseudopregnancy and ovarian follicle numbers. Reprod Domest Anim. 2022;57(12):1636–43. doi: 10.1111/rda.14240 36052807

[84] Kezele P, Skinner MK. Regulation of ovarian primordial follicle assembly and development by estrogen and progesterone: endocrine model of follicle assembly. Endocrinology. 2003;144(8):3329–37. doi: 10.1210/en.2002-0131 12865310

[85] Aiken CE, Tarry-Adkins JL, Penfold NC, Dearden L, Ozanne SE. Decreased ovarian reserve, dysregulation of mitochondrial biogenesis, and increased lipid peroxidation in female mouse offspring exposed to an obesogenic maternal diet. FASEB J. 2016;30(4):1548–56. doi: 10.1096/fj.15-280800 26700734 PMC4799509

[86] Hutt KJ, McLaughlin EA, Holland MK. Primordial follicle activation and follicular development in the juvenile rabbit ovary. Cell Tissue Res. 2006;326(3):809–22. doi: 10.1007/s00441-006-0223-3 16830146

[87] Murdoch WJ, Van Kirk EA, Vonnahme KA, Ford SP. Ovarian responses to undernutrition in pregnant ewes, USA. Reprod Biol Endocrinol. 2003;1:6. doi: 10.1186/1477-7827-1-6 12646075 PMC151801

[88] Norris DO, Lopez KH. Hormones and reproduction of vertebrates. San Diego: Academic Press. 2011.

[89] Fleming R, Jenkins J. The source and implications of progesterone rise during the follicular phase of assisted reproduction cycles. Reprod Biomed Online. 2010;21(4):446–9. doi: 10.1016/j.rbmo.2010.05.018 20800548

[90] Kolibianakis EM, Bourgain C, Papanikolaou EG, Camus M, Tournaye H, Van Steirteghem AC, et al. Prolongation of follicular phase by delaying hCG administration results in a higher incidence of endometrial advancement on the day of oocyte retrieval in GnRH antagonist cycles. Hum Reprod. 2005;20(9):2453–6. doi: 10.1093/humrep/dei069 15890735

[91] Yang Y, Liu B, Wu G, Yang J. Exploration of the value of progesterone and progesterone/estradiol ratio on the hCG trigger day in predicting pregnancy outcomes of PCOS patients undergoing IVF/ICSI: a retrospective cohort study. Reprod Biol Endocrinol. 2021;19(1):184. doi: 10.1186/s12958-021-00862-6 34893087 PMC8665570

[92] Elnashar AM. Progesterone rise on the day of HCG administration (premature luteinization) in IVF: an overdue update. J Assist Reprod Genet. 2010;27(4):149–55. doi: 10.1007/s10815-010-9393-8 20177771 PMC2854984

[93] Lee F-K, Lai T-H, Lin T-K, Horng S-G, Chen S-C. Relationship of progesterone/estradiol ratio on day of hCG administration and pregnancy outcomes in high responders undergoing in vitro fertilization. Fertil Steril. 2009;92(4):1284–9. doi: 10.1016/j.fertnstert.2008.08.024 18829018

[94] Lai T-H, Lee F-K, Lin T-K, Horng S-G, Chen S-C, Chen Y-H, et al. An increased serum progesterone-to-estradiol ratio on the day of human chorionic gonadotropin administration does not have a negative impact on clinical pregnancy rate in women with normal ovarian reserve treated with a long gonadotropin releasing hormone agonist protocol. Fertil Steril. 2009;92(2):508–14. doi: 10.1016/j.fertnstert.2008.06.036 18701101

[95] Lin Y-J, Lan K-C, Huang F-J, Lin P-Y, Chiang H-J, Kung F-T. Reproducibility and clinical significance of pre-ovulatory serum progesterone level and progesterone/estradiol ratio on the day of human chorionic gonadotropin administration in infertile women undergoing repeated in vitro fertilization cycles. Reprod Biol Endocrinol. 2015;13:41. doi: 10.1186/s12958-015-0037-9 25967104 PMC4438509

[96] Mahran A, Khairy M, Elkhateeb R, Hegazy AR, Abdelmeged A, Batiha GE-S, et al. The value of serum progesterone level on day of human chorionic gonadotrophin administration / metaphase II oocyte ratio in predicting IVF/ICSI outcome in patients with normal ovarian reserve. J Ovarian Res. 2021;14(1):52. doi: 10.1186/s13048-021-00800-5 33794989 PMC8017661

[97] Lonergan P, Khatir H, Piumi F, Rieger D, Humblot P, Boland MP. Effect of time interval from insemination to first cleavage on the developmental characteristics, sex ratio and pregnancy rate after transfer of bovine embryos. J Reprod Fertil. 1999;117(1):159–67. doi: 10.1530/jrf.0.1170159 10645257

[98] Sui S, He B, Jia Y, Li R, Cai D, Li X, et al. Maternal protein restriction during gestation and lactation programs offspring ovarian steroidogenesis and folliculogenesis in the prepubertal gilts. J Steroid Biochem Mol Biol. 2014;143:267–76. doi: 10.1016/j.jsbmb.2014.04.010 24787658

[99] Guzmán C, García-Becerra R, Aguilar-Medina MA, Méndez I, Merchant-Larios H, Zambrano E. Maternal protein restriction during pregnancy and/or lactation negatively affects follicular ovarian development and steroidogenesis in the prepubertal rat offspring. Arch Med Res. 2014;45(4):294–300. doi: 10.1016/j.arcmed.2014.05.005 24819035

[100] Khorram O, Keen-Rinehart E, Chuang T-D, Ross MG, Desai M. Maternal undernutrition induces premature reproductive senescence in adult female rat offspring. Fertil Steril. 2015;103(1):291-8.e2. doi: 10.1016/j.fertnstert.2014.09.026 25439841 PMC4282592

[101] Long NM, Nijland MJ, Nathanielsz PW, Ford SP. The effect of early to mid-gestational nutrient restriction on female offspring fertility and hypothalamic-pituitary-adrenal axis response to stress. J Anim Sci. 2010;88(6):2029–37. doi: 10.2527/jas.2009-2568 20190172

[102] Hu W. The role of p53 gene family in reproduction. Cold Spring Harb Perspect Biol. 2009;1(6):a001073. doi: 10.1101/cshperspect.a001073 20457559 PMC2882126

[103] Haraguchi H, Hirota Y, Saito-Fujita T, Tanaka T, Shimizu-Hirota R, Harada M, et al. Mdm2-p53-SF1 pathway in ovarian granulosa cells directs ovulation and fertilization by conditioning oocyte quality. FASEB J. 2019;33(2):2610–20. doi: 10.1096/fj.201801401R 30260703

[104] Cherian-Shaw M, Das R, Vandevoort CA, Chaffin CL. Regulation of steroidogenesis by p53 in macaque granulosa cells and H295R human adrenocortical cells. Endocrinology. 2004;145(12):5734–44. doi: 10.1210/en.2004-0253 15331571

[105] Salehi E, Aflatoonian R, Moeini A, Yamini N, Asadi E, Khosravizadeh Z, et al. Apoptotic biomarkers in cumulus cells in relation to embryo quality in polycystic ovary syndrome. Arch Gynecol Obstet. 2017;296(6):1219–27. doi: 10.1007/s00404-017-4523-5 28988321

[106] Sirotkin AV, Ovcharenko D, Benco A, Mlyncek M. Protein kinases controlling PCNA and p53 expression in human ovarian cells. Funct Integr Genomics. 2009;9(2):185–95. doi: 10.1007/s10142-008-0102-y 19067003

[107] Velazquez MA. Impact of maternal malnutrition during the periconceptional period on mammalian preimplantation embryo development. Domest Anim Endocrinol. 2015;51:27–45. doi: 10.1016/j.domaniend.2014.10.003 25498236

[108] Keim AL, Chi MM, Moley KH. Hyperglycemia-induced apoptotic cell death in the mouse blastocyst is dependent on expression of p53. Mol Reprod Dev. 2001;60(2):214–24. doi: 10.1002/mrd.1080 11553921

[109] Chang T-S, Cho C-S, Park S, Yu S, Kang SW, Rhee SG. Peroxiredoxin III, a mitochondrion-specific peroxidase, regulates apoptotic signaling by mitochondria. J Biol Chem. 2004;279(40):41975–84. doi: 10.1074/jbc.M407707200 15280382

[110] Cree LM, Hammond ER, Shelling AN, Berg MC, Peek JC, Green MP. Maternal age and ovarian stimulation independently affect oocyte mtDNA copy number and cumulus cell gene expression in bovine clones. Hum Reprod. 2015;30(6):1410–20. doi: 10.1093/humrep/dev066 25820694

[111] Ogawa K, Itami N, Ueda M, Kansaku K, Shirasuna K, Kuwayama T, et al. Non-esterified fatty acid-associated ability of follicular fluid to support porcine oocyte maturation and development. Reprod Med Biol. 2018;17(2):155–63. doi: 10.1002/rmb2.12084 29692673 PMC5902458

[112] Herrick JR, Brad AM, Krisher RL. Chemical manipulation of glucose metabolism in porcine oocytes: effects on nuclear and cytoplasmic maturation in vitro. Reproduction. 2006;131(2):289–98. doi: 10.1530/rep.1.00835 16452722

[113] Tsutsumi O, Satoh K, Taketani Y, Kato T. Determination of enzyme activities of energy metabolism in the maturing rat oocyte. Mol Reprod Dev. 1992;33(3):333–7. doi: 10.1002/mrd.1080330315 1449800

[114] Wang L, Lin J, Huang J, Wang J, Zhao Y, Chen T. Selection of ovine oocytes by brilliant cresyl blue staining. J Biomed Biotechnol. 2012;2012:161372. doi: 10.1155/2012/161372 22675245 PMC3366259

[115] Lamas-Toranzo I, Pericuesta E, Bermejo-Álvarez P. Mitochondrial and metabolic adjustments during the final phase of follicular development prior to IVM of bovine oocytes. Theriogenology. 2018;119:156–62. doi: 10.1016/j.theriogenology.2018.07.007 30015144

[116] Nourani MR, Owada Y, Kitanaka N, Sakagami H, Hoshi H, Iwasa H, et al. Occurrence of immunoreactivity for adipocyte-type fatty acid binding protein in degenerating granulosa cells in atretic antral follicles of mouse ovary. J Mol Histol. 2005;36(8–9):491–7. doi: 10.1007/s10735-006-9024-y 16733794

